# Sociodemographic and training profile of nursing professionals in the state of São Paulo in relation to Integrative and Complementary Health Practices

**DOI:** 10.1590/1518-8345.7144.4203

**Published:** 2024-09-23

**Authors:** Juliana Rizzo Gnatta, Thiago da Silva Domingos, Edilaine Cristina da Silva Gherardi-Donato, Suzimar de Fátima Benato Fusco, Leonice Fumiko Sato Kurebayashi, Talita Pavarini Borges

**Affiliations:** 1 Universidade de São Paulo, Escola de Enfermagem, Departamento Médico-Cirúrgico, São Paulo, SP, Brazil; 2 Brazilian Ctr Evidence Based Healthcare, JBI Ctr Excellence JBI Brazil, Sao Paulo, SP, Brazil; 3 Universidade Federal de São Paulo, Escola Paulista de Enfermagem, Departamento de Enfermagem Clínica e Cirúrgica, São Paulo, SP, Brazil; 4 Universidade de São Paulo, Escola de Enfermagem de Ribeirão Preto, Departamento de Enfermagem Psiquiátrica e Ciências Humanas, Centro Colaborador de la OPS/OMS para el Desarrollo de la Investigación en Enfermería, Ribeirão Preto, SP, Brazil; 5 Universidade Estadual de Campinas, Faculdade de Enfermagem, Departamento de Enfermagem Médico-Cirúrgica, Campinas, SP, Brazil; 6 Associação Brazileira de Enfermeiros de Centro Cirúrgico, Recuperação Anestésica e Centro de Material e Esterilização, Comitê de Ensino e Pesquisa em Enfermagem Perioperatória, São Paulo, SP, Brazil; 7 Universidade de São Paulo, Escola de Enfermagem, São Paulo, SP, Brazil; 8 Instituto de Terapia Integrada e Oriental, São Paulo, SP, Brazil; 9 Conselho Regional de Enfermagem de São Paulo, Grupo de Trabalho em Práticas Integrativas e Complementares em Saúde, São Paulo, SP, Brazil; 10 Instituto Pavarini, São Paulo, SP, Brazil

**Keywords:** Nursing, Nurse’s Role, Professional Training, Job Description, Complementary Therapies, Complementary Therapeutic Methods

## Abstract

**Objective:**

to analyze the sociodemographic and training profile of nursing professionals in the state of São Paulo in relation to Integrative and Complementary Practices in Health.

**Method:**

cross-sectional study, developed with 3,794 nursing professionals in the state of São Paulo, from 645 municipalities. To collect data, an online questionnaire was used containing sociodemographic and professional training variables. A hierarchical multiple Poisson regression model was constructed, considering training in practices as the dependent variable, with a significance level of 5%.

**Results:**

of the 3,794 (100%) participants, 479 (12.62%) had training in Integrative and Complementary Health Practices. The most frequent practices were auriculotherapy, Traditional Chinese Medicine/acupuncture and *Reiki.* The predominant training modalities were free, face-to-face and theoretical-practical courses. The variables age, no formal religion, higher education and specialization level, working hours and knowledge and previous experience on integrative practices were significant.

**Conclusion:**

the results indicate the need to encourage and expand the training of Integrative and Complementary Health Practices for nursing professionals, in order to strengthen their performance in health services.

## Highlights

**(1)** 39% of Nursing professionals are unaware of ICHPs.

**(2)** Low proportion (14.5%) of Nursing professionals with ICHPS training.

**(3)** Auriculotherapy, TCIM/acupuncture and Reiki are the most frequent trainings.

**(4)** Free, face-to-face and theoretical-practical courses are predominant during training.

**(5)** There is a large discrepancy in the training hours in ICHPS.

## Introduction

 Integrative and Complementary Health Practices (ICHPS) are therapeutic resources used in a complementary way to conventional treatment, alone or as part of medical rationalities, which are complex medical systems ^(^ 1 ^)^ composed of integrated and structured knowledge and practices. They have an interdisciplinary character with a focus on health promotion and prevention of chronic diseases through changes in lifestyle and self-care ^(^ 1 ^)^ . ICHPS move towards a new paradigm in health, whose main focus is health, through the individual’s search for balance in relation to the natural and social environment to which they belong. 

 In 2013, the World Health Organization ^(^ 2 ^)^ (WHO) adopted in the WHO Strategy document on traditional medicine 2014-2023, which was extended until 2025 ^(^ 2 ^)^ , the name Traditional, Complementary and Integrative Medicines (TCIM) for modalities that encompass ^(^ 1 ^)^ medical rationalities such as Traditional Chinese Medicine, Indian Ayurveda, homeopathy and Anthroposophic Medicine, among others, and ^(^ 2 ^)^ therapeutic practices, which include mind and body interventions, body manipulation, natural and energetic therapies ^(^ 1 ^,^ 3 ^)^ . The different therapeutic practices were called ICHPS in Brazil, with the approval by the Ministry of Health of the National Policy on Integrative and Complementary Practices (NPICP) in the Unified Health System (SUS) in 2006 ^(^ 4 ^)^ . 

 In 2018, the set of practices reached 29 therapeutic modalities recognized by the Ministry of Health and offered by the SUS ^(^ 5 ^)^ . The principles that govern ICHPS are congruent with those of nursing, as they are based on a human-centered vision to offer a comprehensive care structure that can be offered in all clinical environments in order to promote health and well-being, this concept being called integrative nursing ^(^ 6 ^)^ . 

 Holistic and Complementary Therapies were affirmed as a nursing specialty through Resolution of the Federal Nursing Council (Cofen) No. 581 of 2018 ^(^ 7 ^)^ , ensuring support for nursing professionals to work in this scenario, as well as to develop research in the area of ICHPS. 

 There has been an increasing use of ICHPS in the world ^(^ 6 ^)^ and in Brazil during the last decade, reaching a prevalence of 5.2% of the population over 18 years of age ^(^ 8 ^)^ . It is observed that the North and South regions had a higher prevalence of use among a population characterized by female, white colour/race, with higher income, education and age group. Acupuncture, homeopathy, medicinal plants, meditation and yoga were the most prevalent ICHPS in the country ^(^ 8 ^)^ . 

 Numerically, nursing is a category that has national representation among health professions, with approximately 2,873,087 professionals including nurses, nursing technicians, assistants and midwives. The state of São Paulo has 735,296 professionals ^(^ 9 ^)^ , representing around 26% of the Brazilian nursing contingent. 

 There is still a long way to go to strengthen the use of ICHPS by nurses. The Federal Nursing Council has played a fundamental role in defending ICHPS in the SUS, in order to regulate, strengthen, stimulate and disseminate different practices for the benefit of users, bringing greater autonomy and therapeutic resources to Nursing ^(^ 10 ^-^ 12 ^)^ . 

 In relation to São Paulo, the “Technical Chambers” of the Regional Nursing Council of the State of São Paulo (Coren-SP) established a ICHPS Working Group, to study the regulation of nurses’ work in this area, to carry out a survey of those who have already work, subsidize new proposals, substantiating opinions on practices for the category ^(^ 13 ^)^ . 

 The provision of ICHPS in PHC by Nursing in the state of São Paulo was recovered in an article published in 2022, highlighting the growing record of these procedures which, however, had a significant decline during the COVID-19 pandemic ^(^ 14 ^)^ . Records of ICHPS application by nurses in official health information systems (DATASUS) confirm nursing performance, with no data in the scientific literature that provide guidance regarding the profile and preparation of these professionals for the procedures performed. 

 Different formats of ICHPS training have been offered to professionals. Free courses have been offered by the Ministry of Health in partnership with universities for higher education professionals who work in PHC ^(^ 15 ^)^ . In addition to these, public and private institutions have offered free and paid courses for training in the different ICHPS, and there is no official mapping that brings together these initiatives. In view of the above, the present study sought to fill a gap in knowledge about how the ICHPS carried out especially by Brazilian nursing and in different states have been implemented, whether in the care or training context. Thus, the objective was to analyze the sociodemographic and training profile of nursing professionals in the state of São Paulo in relation to Integrative and Complementary Practices in Health. 

## Method

### Study design

 Observational, cross-sectional and descriptive study. This study was guided by the STROBE guideline - The Strengthening the Reporting of Observational Studies in Epidemiology ^(^ 16 ^)^ . 

### Setting

The research covered nursing professionals in the state of São Paulo who had active registration with Coren-SP during the study period.

### Period

 The research was carried out from July 27 ^th^ to September 17 ^th^ , 2022. 

### Population

The population consisted of nursing professionals (assistants, technicians, nurses and midwives) who work in the state of São Paulo.

### Selection criteria

The inclusion criteria were: active nursing professional registered at Coren-SP, at least 18 years old and with internet access, of both sexes. Those professionals who did not answer all the questions in the data collection questionnaire were excluded from the sample.

### Sample definition

Calculation of the sample size with an unknown universe and prevalence (50%) and standardized errors (2% alpha and 20% beta), without assuming errors and failures, resulting in an expected sample of at least 2396 volunteers.

### Recruitment

The recruitment of nursing professionals took place online. The research was widely publicized in the virtual media most used by the target population, with links to the questionnaire being posted in various open virtual environments such as: University website, Coren-SP website, social networks (Whatsapp, Instagram and Facebook) and emails, with the possibility of sharing means of dissemination to expand reach. Additionally, all professionals with an active professional license in Coren-SP received an invitation to participate in the study via email from the council itself. In the body of the email, an invitation message was sent with information about the research and participation, ending with a link that directed the professional to the electronic Informed Consent Form. On this page, after reading the explanations about the research and ethical aspects, those who agreed to participate expressed their agreement through the electronic consent form.

In this way, 6208 people began the research, 77 of whom refused to participate and 2337 did not complete the questionnaire completely, representing 38.88% of losses. Therefore, the sample consisted of 3,794 nursing professionals from 645 municipalities in the state of São Paulo.

### Data collection

To collect data, a self-administered online questionnaire was used, developed by the study authors (supplementary material). Data collection took place using the Research Electronic Data Capture (REDCap) research tool, with the purpose of assisting the study data collection and management process.

The questionnaire was built in REDCap to ensure that only one response per participant was considered in data analysis, requesting the participant’s email when filling out and only one response with duplicate emails was validated.

### Study variables

The variables of interest to achieve the study objectives were demographic variables (age, gender, race/colour, marital status, family income, religion, city of residence) and those related to professional training (professional category, training time, time of work in nursing and ICHPS, place of work in nursing and ICHPS, specialty in nursing, knowledge and interest in ICHPS, training in ICHPS).

### Data processing and analysis

The data were exported from REDCap directly to a structured Microsoft Excel spreadsheet, using the platform’s own automatic procedures, and analysed using the Statistical Package for the Social Sciences (SPSS) statistical program.

Descriptive and inferential statistics were performed. The sample characterization was carried out based on exploratory analysis using means, frequency and percentage. For comparisons between participants who did and did not undergo ICHPS training, the Mann-Whitney test was applied. Data distribution was assessed using the Shapiro-Wilk test. To evaluate the associations between the variable referring to ICHPS training and the other qualitative variables, Pearson’s Chi-square test was applied.

A hierarchical modified multiple Poisson regression model was constructed, with robust variance, considering ICHPS training as the dependent variable. The results presented the prevalence ratio estimates obtained, as well as their respective confidence intervals and p-values. To carry out the analyses, the statistical software SAS version 9.4 was used and a significance level of 5% was considered.

### Ethical aspects

Ethical standards and guidelines were respected and the project was approved by the Research Ethics Committee of the Ribeirão Preto School of Nursing at the University of São Paulo (EERP/USP) (CAAE 55755821.0.0000.5393). The project complies with Resolution 466/12.

## Results

 Out of the 3,794 (100%) participants, the majority were women (86.95%), with mean age 40.11 (SD 9.68) years, with 12.01 (SD 8.90) years of training and 11.77 (SD 9.26) years of professional experience; 479 professionals are trained in ICHPS, which corresponds to 12.62% of respondents. The distribution of sociodemographic and training variables of nursing professionals who have or do not have training in ICHPS are presented in Table 1 . Categorical variables are expressed in numbers (percentages) and continuous variables are expressed as mean (standard deviation). 

Table 2 shows the characteristics of the experience with ICHPS by nursing professionals (n = 3794) with and without ICHPS training. It is observed that 93.95% of professionals with training in ICHPS had some previous experience with ICHPS, practical or theoretical-practical, mainly in the institution where they work. This experience was related to the quality of users of health services or as health professionals. Categorical variables are expressed in numbers (percentages) and continuous variables are expressed as means (standard deviation). 

**Table 1 -:** Distribution of sociodemographic and training variables of nursing professionals with and without training in Integrative and Complementary Health Practices in the state of São Paulo (N* = 3794). São Paulo, Brazil, 2022

**Variables**	**No (3315)**		**Yes (479)**		**p-value**
Age	39.68	(9.57)	43.05	(9.92)	<0.0001 ^†^
Gender					0.2177 ^‡^
Female	2874	87.12	425	12.88	
Male	441	89.09	54	10.91	
Race/Colour					< 0.0001 ^‡^
Yellow/White/Indigenous	1965	85.47	334	14.53	
Brown/Black	1350	90.30	145	9.70	
Marital status					0.2013 ^‡^
Without partner	1044	88.40	137	11.60	
With partner	2271	86.91	342	13.09	
Family Income					< 0.0001 ^‡^
Up to 2 minimum wages	770	94.59	44	5.41	
Between 2 to 4 minimum wages	1293	91.18	125	8.82	
Between 4 to 10 minimum wages	1053	81.75	235	18.25	
Above 10 minimum wages	199	72.63	75	27.37	
Religion					0.0639 ^‡^
No	393	84.70	71	15.30	
Yes	2922	87.75	408	12.25	
Regions					0.4090 ^‡^
São Paulo	2079	87.35	301	12.65	
Sorocaba/Campinas/São José dos Campos	712	86.51	111	13.49	
Bauru/Marília/Presidente Prudente/Araçatuba	223	90.65	23	9.35	
São José do Rio Preto/Ribeirão Preto/Araraquara	301	87.25	44	12.75	
Academic background					< 0.0001 ^‡^
Vocational	1300	96.58	46	3.42	
Bachelor`s degree	760	92.57	61	7.43	
*Lato sensu* (professional)	1104	79.37	287	20.63	
*Stricto sensu* (academic)	151	63.98	85	36.02	
Professional training in Nursing (years)	11.48	8.62	15.72	9.85	< 0.0001 ^†^
Years of professional experiences in Nursing	11.20	9.01	15.73	9.98	< 0.0001 ^†^
Weekly workload					< 0.0001 ^‡^
<= 40 hours	2341	85.56	395	14.44	
> 40 hours	974	92.06	84	7.94	
Employment bond					0.7990 ^‡^
1	2164	87.12	320	12.88	
2 or more	502	88.07	68	11.93	
Without one	649	87.70	91	12.30	

Categorical variables are expressed in numbers (percentages) and continuous variables are expressed as means (standard deviation). No: No training in Integrative and Complementary Health Practices (ICHPS); Yes: trained in ICHPS

* N = Number of participants analyzed

† Mann-Whitney test

‡ Chi-square tests

**Table 2 -:** Characteristics of the experience with Integrative and Complementary Health Practices (ICHPS) of nursing professionals with and without training in ICHPS in the state of São Paulo (N* = 3794). São Paulo, Brazil, 2022

**Variables**	**No (3315)**		**Yes (479)**		**p-value†**
Informed to know what ICHPS are					< 0.0001
Yes	1842	79.64	471	20.36	
No	1473	99.46	8	0.54	
Living or experience with ICHPS					< 0.0001
Yes	988	68.71	450	31.29	
No	2327	98.77	29	1.23	
Experience					<0.0001
Theoretical	375	87.01	56	12.99	
Practical	453	70.67	188	29.33	
Theoretical and practical	160	43.72	206	56.28	
Has no experience	2327	98.77	29	1.23	
Role in this first experience:					<0.0001
User (own usage)	230	65.71	120	34.29	
Family member, friends	79	91.86	7	8.14	
Student (during their training in Nursing)	248	81.05	58	18.95	
Student (Other academic background besides nursing)	27	21.95	96	78.05	
Health professional (contact in the institution where they belong)	404	70.51	169	29.49	
Has no experience					

Categorical variables are expressed in numbers (percentages) and continuous variables are expressed as means (standard deviation). No: No training in ICHPS; Yes: With training in ICHPS. *N = Number of participants analyzed

† p= Significance level analyzed by the chi-square test

 Based on the characteristics of the experience with ICHPS by nursing professionals presented, a model of the profile of nursing professionals with training in ICHPS was constructed ( Table 3 ). The variables age, not having a formal religion, higher education and specialized training ( *lato* - professional and/or *stricto sensu* - academic), working a workload equal to or less than 40 hours per week, informing what ICHPS are presented statistical significance. 

**Figure 1 -:**
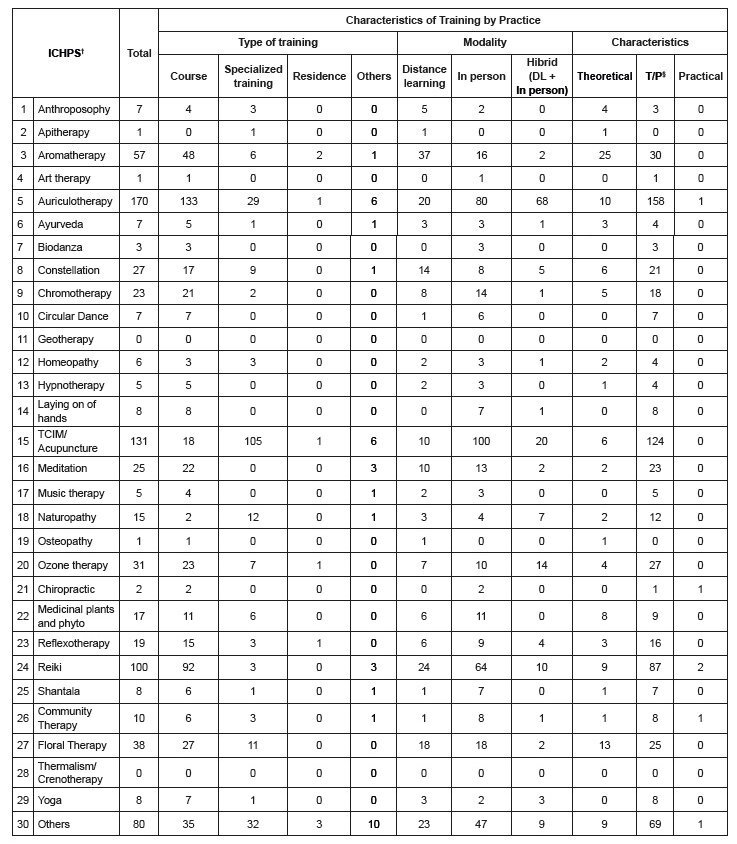
Self-reported characteristics of training courses in Integrative and Complementary Health Practices carried out by nursing professionals in the state of São Paulo (N* = 479). São Paulo, Brazil, 2022

 The characteristics of ICHPS training by nursing professionals in the state of São Paulo are presented in Figure 1 . The most identified practices were auriculotherapy, Traditional Chinese Medicine/acupuncture and *Reiki* . Regarding the characteristics of the courses carried out for training, open course modalities (professional education with reduced workload, such as workshops), face-to-face and theoretical-practical courses prevailed. 

 The workload represents the number of hours dedicated to theoretical-practical training in each course, offering an overview of the time needed to acquire knowledge and skills in each of the therapeutic approaches. Acupuncture, naturopathy and community therapy stand out as those with the highest average training hours and biodance, art therapy and osteopathy, with the lowest training hours. The average values presented in Table 4 regarding the course load in ICHPS showed a significantly high standard deviation, indicating that there is considerable variation in the course load offered, even within the same ICHPS modality. 

**Table 3 -:** Profile of nursing professionals trained in Integrative and Complementary Health Practices in the state of São Paulo (N* = 3794). São Paulo, Brazil, 2022

**Variables**	**Step 1**				**Step 2**				**Step 3**			
	**PR** ^†^	**C.I.** ^‡^ **(95%)**		**p-value** ^¶^	**RP** ^†^	**C.I.** ^‡^ **(95%)**		**p-value** ^¶^	**RP** ^†^	**C.I.** ^‡^ **(95%)**		**p-value** ^¶^
		**L.L.** ^§^	**U.L.** ^||^			**L.L.** ^§^	**U.L.** ^||^			**L.L.** ^§^	**U.L.** ^||^	
Age	1.02	1.01	1.03	**< 0.0001**	1.02	1.01	1.02	**0.0002**	1.01	1.01	1.02	**0.0004**
Female Gender	1.23	0.95	1.60	0.1219								
Race/Colour (Yellow/White/Indigenous)	1.21	1.01	1.46	**0.0389**	1.10	0.92	1.32	0.3036				
Family Income (Between 2 to 4 minimum wages**)	1.56	1.12	2.18	**0.0084**	1.08	0.78	1.51	0.6378	1.09	0.79	1.49	0.6048
Family Income (Between 4 to 10 minimum wages**)	3.02	2.21	4.13	**< 0.0001**	1.30	0.94	1.79	0.1129	1.15	0.84	1.58	0.3680
Family Income (Above 10 minimum wages**)	4.16	2.90	5.96	**< 0.0001**	1.44	1.00	2.08	0.0505	1.16	0.82	1.63	0.4105
Religion (No)	1.27	1.01	1.60	**0.0373**	1.25	1.01	1.56	**0.0416**	1.23	1.02	1.48	**0.0342**
Education (B.A.)					1.97	1.35	2.88	**0.0004**	1.46	1.02	2.09	**0.0404**
Education ( *Lato sensu* – professional training)					4.88	3.54	6.71	**< 0.0001**	2.77	2.03	3.78	**< 0.0001**
Education ( *Stricto sensu* – academic training)					7.39	5.16	10.58	**< 0.0001**	3.32	2.36	4.66	**< 0.0001**
Weekly workload (<= 40 hours)					1.57	1.26	1.96	**< 0.0001**	1.40	1.14	1.71	**0.0013**
Informed they know what ICHPS ^††^ are (Yes)									4.84	2.35	10.01	**< 0.0001**
Living or experience with ICHPS ^††^ (Yes)									10.47	7.18	15.28	**< 0.0001**

* N = Number of participants analyzed

† PR = Prevalence ratio. The probability of presenting the result “Yes” was estimated

‡ C.I. = Confidence interval

§ L.L. = Lower limit

|| U.L. = Upper limit

¶ p-value = Significance level from the hierarchical modified multiple Poisson Regression Model, with robust variance

** Current minimum wage = R$1,212.00, Brazil, 2022

†† ICHPS = Integrative and Complementary Practices in Health

*N = Number of participants analyzed; ^†^ ICHPS = Integrative and Complementary Practices in Health; ^‡^ DL = Distance learning; ^§^ T/P = Theoretical/Practical

**Table 4 -:** Self-reported workload of training courses in Integrative and Complementary Practices in Health carried out by nursing professionals in the State of São Paulo (N* = 479). São Paulo Brazil, 2022

**ICHPS**	N*	**Mean**	**Standard deviation**	**Minimum**	**Q1** ^†^	**Median**	**Q3** ^‡^	**Maximum**
Anthroposophy	7	167.14	180.44	30.00	40.00	120.00	220.00	540.00
Apitherapy	1	56.00	-	-	-	-	-	-
Aromatherapy	56	159.80	273.83	5.00	20.00	40.00	180.00	1744.00
Art therapy	1	30.00	-	-	-	-	-	-
Auriculotherapy	165	124.45	211.10	0.00	30.00	75.00	100.00	1300.00
Ayurveda	6	298.67	401.93	12.00	60.00	200.00	220.00	1100.00
Biodanza	3	10.00	8.66	5.00	5.00	5.00	20.00	20.00
Constellation	27	232.22	223.69	0.00	80.00	150.00	360.00	900.00
Chromotherapy	22	59.64	77.67	2.00	30.00	40.00	60.00	360.00
Circular Dance	7	94.00	137.77	16.00	20.00	40.00	90.00	400.00
Geotherapy	0	-	-	-	-	-	-	-
Homeopathy	6	400.00	301.70	42.00	58.00	450.00	600.00	800.00
Hypnotherapy	5	42.00	30.92	0.00	26.00	40.00	72.00	72.00
Laying on of hands	7	64.57	44.22	20.00	24.00	50.00	120.00	120.00
TCIM/Acupuncture	125	860.59	584.22	3.00	380.00	800.00	1200.00	3500.00
Meditation	24	94.54	86.94	2.00	30.00	50.00	170.00	300.00
Music therapy	4	35.00	32.72	4.00	12.00	28.00	58.00	80.00
Naturopathy	13	748.46	777.49	30.00	460.00	480.00	480.00	3000.00
Osteopathy	1	30.00	-	-	-	-	-	-
Ozone therapy	31	218.32	382.79	20.00	120.00	120.00	200.00	2200.00
Chiropractic	1	40.00	-	-	-	-	-	-
Medicinal plants and phyto	17	221.29	214.61	8.00	60.00	120.00	360.00	720.00
Reflexotherapy	19	252.89	458.21	8.00	40.00	100.00	288.00	2000.00
Reiki	93	125.37	394.17	1.00	16.00	30.00	60.00	2400.00
Shantala	8	39.25	58.38	8.00	8.00	19.00	36.00	180.00
Community Therapy	10	409.80	583.06	8.00	30.00	210.00	500.00	1940.00
Floral Therapy	37	191.73	234.68	4.00	30.00	90.00	360.00	1200.00
Thermalism/Crenotherapy	0	-	-	-	-	-	-	-
Yoga	7	259.14	212.88	14.00	120.00	180.00	500.00	600.00
Others	74	398.42	655.09	0.00	40.00	135.00	360.00	4138.00

* N = Number of participants analyzed

† Q1 = First quartile

‡ Q3 = Third quartile

## Discussion

The population trained in ICHPS had an average age of 43.05 years, with a predominance of females, white, yellow and indigenous race/color, living with partners, without declaration of religion, income between 4 and 10 minimum wages, with specialized higher education and, on average, 15 years of training and professional experience. A significant difference was observed in terms of average age, length of training, and professional experience, being greater among the population that had training in ICHPS.

 Regarding knowledge, 39% (n=1481) reported lack of knowledge about ICHPS. A similar result was identified among Iranian nurses, whose knowledge about ICHPS was assessed as low ^(^ 17 ^)^ . A mapping study that worked with a convenience sample with feedback from ten European countries did not find a consistent approach to training nurses in ICHPS, as despite courses being taught in regular educational institutes, they are not integrated into regular undergraduate education ^(^ 18 ^)^ . Although the findings of this study are convergent with the aforementioned study, the interpretation of the data must consider the historical-cultural characteristics of different countries regarding medical rationalities and traditional healing systems, as well as the characteristics of training policies and the performance of professionals. in ICHPS. 

 In the present study, professionals with training in ICHPS mentioned that the most frequent practices were auriculotherapy, Traditional Chinese Medicine/acupuncture and reiki. This result is in disagreement with data from the National Health Survey, which shows acupuncture, homeopathy, medicinal plants, meditation and yoga ^(^ 8 ^)^ . Auriculotherapy as the most frequent ICHPS in the studied population may be related to the training that has been offered in Brazil, since 2016, for higher education professionals who work in PHC. In these courses, the professionals who participate are female, nursing professionals with an average age of 36.8 years ^(^ 19 ^)^ . This profile is similar to the nursing professionals in the state of São Paulo investigated in the present study. When compared to European countries, it was observed that the main ICHPS offered for nursing training are massage, meditation, mindfulness and relaxation techniques ^(^ 18 ^)^ . Training, whether at a national or global level, represents a gap that deserves efforts to expand access to training for health professionals, especially nursing professionals, taking into account the results of international and national investigations that indicate the lack of knowledge as one of the main weaknesses for expansion of ICHPS offerings ^(^ 17 ^,^ 20 ^)^ . This finding puts into perspective the discussion about the initial and continuing training of Nursing professionals with regard to ICHPS, as it is observed that there is a lack of a consistent approach in both Brazil and Europe in the education of nurses in ICHPS and integrative nursing ^(^ 18 ^)^ . For European nursing, as with Brazilian nursing, courses are taught. However, there is no national guideline that integrates ICHPS into the regular teaching curriculum ^(^ 18 ^)^ . 

 In the present study, it was found that professionals with some training in ICHPS also had previous contact with some practice, either during their training as a professional or in the institution in which they work. Thus, the importance of training courses offering subjects in their curricula is highlighted to raise awareness among future professionals about the topic and present ICHPS as an approach to health care. In Brazil, it is observed that degrees in Nursing, Medicine and Dentistry are characterized by offering subjects on ICHPS that are elective, theoretical and not integrated into the curricular matrices ^(^ 21 ^-^ 22 ^)^ . 

 Still in relation to training, the findings reflect the variability in the workload for each of the different training courses. This result corroborates the absence of regulatory guidelines regarding a minimum curriculum, with consequent discrepancies. In this sense, there is an urgent need to establish resolutions that guide the category on the minimum workload for each training for each practice. The results show that professionals understood as “training” both free courses, dissemination courses, lectures, workshops, and training courses, demonstrating a lack of clarity about what a training process represents. Variability in training is a worrying aspect highlighted in the literature, constituting a weakness for the ICHPS area itself, for professional training and for users of health services ^(^ 17 ^)^ . 

 From training to performance, there are also challenges related to the dispute for space between professional categories, institutional culture, and the availability of time and resources to offer ICHPS ^(^ 23 ^)^ . These developments are observed in nursing employment contracts that often do not cover or recognize the provision of ICHPS as part of their professional duties. There are also weaknesses in the management of health services, which makes it difficult to understand, recognize and offer ICHPS, resulting in the lack of availability of material resources and systematized dissemination. This problem directly reflects the discrepancy between the real offer and that recorded in the information systems, highlighting the problem of undersized registration of ICHPS ^(^ 24 ^)^ . 

 On the other hand, training in ICHPS and the supply of services are increasing in the national territory ^(^ 8 ^,^ 21 ^-^ 22 ^)^ . This situation allows us to identify the reasons why Nursing professionals - the largest workforce in the Unified Health System - persist in investing in the training and provision of ICHPS in health services. This reality calls on government entities and professional bodies to analyze the ethical-legal and technical framework that supports the actions. 

 Regarding qualifications, it was found in the investigated population that there was a demand for qualifications in the 29 complementary therapies set out in the NPICP ^(^ 4 ^)^ , however, in the specific Nursing Resolutions, only ten practices are included as specialties of the profession ^(^ 7 ^)^ . The institutional dimension is included in this problem, such as the elaboration and availability of Clinical Protocols and Standard Operating Procedures that enable Nursing to act in the provision of ICHPS in health services. 

 It is noteworthy that ICHPS have gained prominence on the world stage. In 2023, the first Global Summit on Traditional Medicine took place in India, in parallel with the interministerial health meeting composed of members of the Group of 20 (G20). The objective of the summit was to record the political commitment of member states to offering their populations evidence-based Traditional Medicine to meet their health and well-being needs ^(^ 25 ^)^ . As it is a universal health issue prioritized by a global entity, it is up to Nursing to incorporate such practices in the exercise of its profession. 

This study has limitations that refer to the data obtained, especially in relation to training. A considerable standard deviation was observed in the training hours among the study respondents, indicating that lectures were possibly considered by professionals as a training activity. In this sense, it is suggested that in future studies the investigative question that guides this issue is linked to the practice carried out in ICHPS, for example, “How many hours were invested in your training that qualified you to apply this ICHPS”?

The contributions of the study refer to the survey of aspects related to the training and performance of Nursing in the federative unit of the country that has the highest concentration of professionals, responding to an international and national scientific gap regarding the sociodemographic and professional profile of Nursing in ICHPS. Based on the findings, it is argued about the importance of public policies in the area of health and their articulation and information with human resources training policies.

## Conclusion

In the state of São Paulo, although there is a considerable number of nursing professionals who are interested in ICHPS, almost half of the professionals are unaware of these practices. The training of professionals in the area takes place mainly among nurses around forty years of age, with specialization and approximately fifteen years of professional experience.

It is noteworthy that minimum guidelines in relation to training must be considered for the training of Nursing professionals in ICHPS, as well as in other health professions. Standardization is essential for the continuity and expansion of the population’s access to ICHPS, with the guarantee of duly qualified professionals to safely offer therapeutic resources in different institutional settings.
