# Supplementary material for: Sociodemographic and training profile of nursing professionals in the state of São Paulo in relation to Integrative and Complementary Health Practices
Source: Rev Lat Am Enfermagem. 2024 Sep 23;32:e4203. doi: 10.1590/1518-8345.7144.4203 (PMC11421516; doi:10.1590/1518-8345.7144.4203)
Supplement: Translation

# Translation: Perfil sociodemográfico y de formación de los profesionales de enfermería del estado de São Paulo en relación a las Prácticas Integrativas y Complementarias de Salud

**DOI:** 10.1590/1518-8345.7144.4202

**Published:** 2024-09-23

**Authors:** Juliana Rizzo Gnatta, Thiago da Silva Domingos, Edilaine Cristina da Silva Gherardi-Donato, Suzimar de Fátima Benato Fusco, Leonice Fumiko Sato Kurebayashi, Talita Pavarini Borges

**Affiliations:** 1 Universidade de São Paulo, Escola de Enfermagem, Departamento Médico-Cirúrgico, São Paulo, SP, Brasil; 2 Brazilian Ctr Evidence Based Healthcare, JBI Ctr Excellence JBI Brazil, Sao Paulo, SP, Brasil; 3 Universidade Federal de São Paulo, Escola Paulista de Enfermagem, Departamento de Enfermagem Clínica e Cirúrgica, São Paulo, SP, Brasil; 4 Universidade de São Paulo, Escola de Enfermagem de Ribeirão Preto, Departamento de Enfermagem Psiquiátrica e Ciências Humanas, Centro Colaborador de la OPS/OMS para el Desarrollo de la Investigación en Enfermería, Ribeirão Preto, SP, Brasil; 5 Universidade Estadual de Campinas, Faculdade de Enfermagem, Departamento de Enfermagem Médico-Cirúrgica, Campinas, SP, Brasil; 6 Associação Brasileira de Enfermeiros de Centro Cirúrgico, Recuperação Anestésica e Centro de Material e Esterilização, Comitê de Ensino e Pesquisa em Enfermagem Perioperatória, São Paulo, SP, Brasil; 7 Universidade de São Paulo, Escola de Enfermagem, São Paulo, SP, Brasil; 8 Instituto de Terapia Integrada e Oriental, São Paulo, SP, Brasil; 9 Conselho Regional de Enfermagem de São Paulo, Grupo de Trabalho em Práticas Integrativas e Complementares em Saúde, São Paulo, SP, Brasil; 10 Instituto Pavarini, São Paulo, SP, Brasil

**Keywords:** Enfermería, Rol de la Enfermera, Capacitación Profesional, Perfil Laboral, Terapias Complementarias, Métodos Terapéuticos Complementarios

## Abstract

**Objetivo:**

analizar el perfil sociodemográfico y de formación de los profesionales de enfermería del estado de São Paulo en relación a las Prácticas Integrativas y Complementarias en Salud.

**Método:**

estudio transversal, desarrollado con 3.794 profesionales de enfermería del estado de São Paulo, provenientes de 645 municipios. Para la recolección de datos se utilizó un cuestionario *online* que contenía variables sociodemográficas y de formación profesional. Se construyó un modelo de regresión de Poisson múltiple jerárquico, considerando la formación en prácticas como variable dependiente, con nivel de significancia del 5%.

**Resultados:**

de los 3.794 (100%) participantes, 479 (12,62%) tenían formación en Prácticas Integrativas y Complementarias en Salud. Las prácticas más frecuentes fueron la auriculoterapia, la Medicina Tradicional China/acupuntura y el *reiki.* Las modalidades de formación predominantes fueron cursos libres, presenciales y teórico-prácticos. Las variables edad, sin religión formal, nivel superior de formación y especialización, carga horaria de trabajo, y conocimiento y experiencia previa sobre prácticas integrativas presentaron significancia.

**Conclusión:**

los resultados indican la necesidad de incentivar y expandir la formación en Prácticas Integrativas y Complementarias en Salud a los profesionales de enfermería, con el fin de fortalecer su actuación en los servicios de salud.

## Destacados

**(1)** 39% de profesionales de Enfermería desconocen las PICS.

**(2)** Baja proporción (14,5%) de profesionales de Enfermería con formación en PICS.

**(3)** Auriculoterapia, MTC/acupuntura y *reiki* son las formaciones más frecuentes.

**(4)** Cursos libres, presenciales y teórico-prácticos predominan en la formación.

**(5)** Existe una gran discrepancia en la carga horaria de formación en PICS.

## Introducción

 Las Prácticas Integrativas y Complementarias en Salud (PICS) son recursos terapéuticos empleados de forma complementaria al tratamiento convencional, aislados o como parte de las racionalidades médicas, las cuales son sistemas médicos complejos ^(^ 1 ^)^ compuestos por conocimientos y prácticas integradas y estructuradas. Poseen carácter interdisciplinario enfocado en la promoción de la salud y prevención de enfermedades crónicas a partir de cambios en el estilo de vida y autocuidado ^(^ 1 ^)^ . Las PICS se encaminan hacia un nuevo paradigma en salud, cuyo enfoque principal es la salud, mediante la búsqueda de equilibrio del individuo en relación con su entorno natural y social. 

 En 2013, la Organización Mundial de la Salud ^(^ 2 ^)^ (OMS) adoptó en el documento de Estrategia de la OMS sobre medicina tradicional 2014-2023, el cual fue prorrogado hasta 2025 ^(^ 2 ^)^ , la denominación Medicinas Tradicionales, Complementarias e Integrativas (MTCI) para las modalidades que abarcan (1) racionalidades médicas como la Medicina Tradicional China, Ayurveda india, homeopatía y Medicina Antroposófica, entre otras, y (2) prácticas terapéuticas, las cuales incluyen las intervenciones mente y cuerpo, terapias de manipulación corporal, naturales y energéticas ^(^ 1 ^,^ 3 ^)^ . Las diferentes prácticas terapéuticas fueron denominadas en Brasil como PICS, con la aprobación por el Ministerio de Salud de la Política Nacional de Prácticas Integrativas y Complementarias (PNPIC) en el Sistema Único de Salud (SUS) en el año 2006 ^(^ 4 ^)^ . 

 En 2018, el conjunto de prácticas alcanzó 29 modalidades terapéuticas reconocidas por el Ministerio de Salud y ofrecidas por el SUS ^(^ 5 ^)^ . Los principios que rigen las PICS son congruentes con los de la enfermería, pues parten de una visión centrada en el ser humano para ofrecer una estructura de cuidados integrales y que puedan ser ofrecidos en todos los ambientes clínicos a fin de promover la salud y el bienestar, siendo este concepto denominado por enfermería integrativa ^(^ 6 ^)^ . 

 Las Terapias Holísticas y Complementarias fueron afirmadas como especialidad de enfermería por medio de la Resolución del Consejo Federal de Enfermería (Cofen) nº 581 de 2018 ^(^ 7 ^)^ , asegurando el respaldo para la actuación del profesional de enfermería en ese escenario, así como para desarrollar investigaciones en el área de las PICS. 

 Se observa un creciente uso de las PICS en el mundo ^(^ 6 ^)^ y en Brasil durante la última década, alcanzando una prevalencia de 5,2% de la población mayor de 18 años ^(^ 8 ^)^ . Se observa que las regiones Norte y Sur tuvieron mayor prevalencia de uso entre una población caracterizada por el sexo femenino, color/raza blanca, con mayor renta, escolaridad y franja etaria. Acupuntura, homeopatía, plantas medicinales, meditación y yoga fueron las PICS de mayor prevalencia en el país ^(^ 8 ^)^ . 

 Numéricamente, la enfermería es una categoría que tiene representatividad nacional entre las profesiones de la salud, contando con aproximadamente 2.873.087 de profesionales entre enfermeros, técnicos de enfermería, auxiliares y obstetrices. El estado de São Paulo tiene 735.296 profesionales ^(^ 9 ^)^ , representando cerca del 26% del contingente de la enfermería brasileña. 

 Un largo camino aún necesita ser recorrido para fortalecer el uso de las PICS por los enfermeros. El Consejo Federal de Enfermería ha tenido un papel fundamental en la defensa de las PICS en el SUS, en el sentido de regular, fortalecer, estimular y difundir las diferentes prácticas para beneficio de usuarios, trayendo mayor autonomía y recursos terapéuticos para la Enfermería ^(^ 10 ^-^ 12 ^)^ . 

 En lo que respecta a São Paulo, las “Cámaras Técnicas” del Consejo Regional de Enfermería del Estado de São Paulo (Coren-SP) instituyeron un Grupo de Trabajo PICS, para estudiar la regulación de la actuación del enfermero en esta área, hacer un levantamiento de aquellos que ya trabajan, subsidiar nuevas propuestas, fundamentando opiniones sobre las prácticas para la categoría ^(^ 13 ^)^ . 

 La oferta de PICS en la APS por la Enfermería en el estado de São Paulo fue recuperada en un artículo publicado en 2022, evidenciando el registro creciente de estos procedimientos que, sin embargo, tuvo un declive importante durante la pandemia de covid-19 ^(^ 14 ^)^ . Los registros de la aplicación de PICS por enfermeros en los sistemas de información en salud oficiales (DATASUS) confirman la actuación de la enfermería, no habiendo datos en la literatura científica que orienten sobre el perfil y la preparación de estos profesionales para los procedimientos realizados. 

 Diferentes formatos de capacitaciones en PICS han sido ofrecidos para los profesionales. Cursos gratuitos han sido ofrecidos por el Ministerio de Salud en asociación con universidades para los profesionales de nivel superior que actúan en la APS ^(^ 15 ^)^ . Además de estos, instituciones públicas y privadas han ofrecido cursos gratuitos y pagos para la capacitación en las diferentes PICS, no habiendo un mapeo oficial que congregue estas iniciativas. Ante lo expuesto, el presente estudio buscó llenar un vacío de conocimiento sobre cómo se ha dado la inserción de las PICS realizadas especialmente por la enfermería brasileña y en los diferentes estados, sea en el ámbito asistencial o formativo. De esta forma, tuvo como objetivo analizar el perfil sociodemográfico y de formación de los profesionales de enfermería del estado de São Paulo en relación a las Prácticas Integrativas y Complementares en Salud. 

## Método

### Tipo del estudio

 Estudio observacional, transversal y descriptivo. Este estudio fue guiado por la directriz STROBE - *The Strengthening the Reporting of Observational Studies in Epidemiology* ^(^ 16 ^)^ . 

### Escenário

La investigación abarcó a los profesionales de enfermería del estado de São Paulo que poseían el registro de inscripción activa del Coren-SP durante el período del estudio.

### Período

La investigación se llevó a cabo en el período del 27 de julio al 17 de septiembre de 2022.

### Población

La población estuvo constituida por profesionales de enfermería (auxiliares, técnicos, enfermeros y obstetrices) que actúan en el estado de São Paulo.

### Criterios de selección

Se consideraron como criterios de inclusión: profesional de enfermería activo registrado en el Coren-SP, con edad mínima de 18 años y acceso a internet, de ambos sexos. Fueron excluidos de la muestra aquellos profesionales que no respondieron a todas las preguntas del cuestionario de recolección de datos.

### Definición de la muestra

El cálculo del tamaño muestral con universo y prevalencia desconocidos (50%) y errores estandarizados (2% alfa y 20% beta), sin suponer errores y fallas, resultó en una muestra esperada de al menos 2396 voluntarios.

### Reclutamiento

 El reclutamiento de los profesionales de enfermería se llevó a cabo de forma *online* . Se realizó una amplia divulgación de la investigación en los medios virtuales más utilizados por la población objetivo, publicando enlaces del cuestionario en diversos entornos virtuales abiertos como: sitio *web* de universidades, sitio *web* del Coren-SP, redes sociales ( *Whatsapp* , *Instagram* y *Facebook* ) y correos electrónicos, con posibilidad de compartir los medios de divulgación para ampliar el alcance. Adicionalmente, todos los profesionales con licencia profesional activa en el Coren-SP recibieron la invitación para participar en el estudio por medio de un correo electrónico del propio consejo. En el cuerpo del correo se envió un mensaje de invitación con información sobre la investigación y la participación, concluyendo con un enlace que dirigía al profesional al Término de Consentimiento Informado (TCI) electrónico. En esa página, tras leer las explicaciones sobre la investigación y los aspectos éticos, aquellos que aceptaron participar expresaron su acuerdo mediante el TCI electrónico. 

De esta manera, iniciaron la investigación 6208 personas, de las cuales 77 se negaron a participar y 2337 no completaron el cuestionario en su totalidad, representando el 38,88% de pérdidas. Por lo tanto, la muestra estuvo constituida por 3.794 profesionales de enfermería de 645 municipios del estado de São Paulo.

### Recolección de datos

 Para la recolección de datos se utilizó un cuestionario en línea autoaplicable, desarrollado por los autores del estudio (material suplementario). La recolección de datos se llevó a cabo mediante la herramienta de investigación *Research Electronic Data Capture* (REDCap), con el propósito de asistir el proceso de recolección y gestión de los datos del estudio. 

El cuestionario se construyó en REDCap para asegurar que se considerara solo una respuesta por participante en el análisis de los datos, solicitándose el correo electrónico del participante en el acto del llenado y solo una respuesta con correos electrónicos duplicados fue validada.

### Variables del estudio

Las variables de interés para alcanzar los objetivos del estudio fueron las variables demográficas (edad, sexo, raza/color, estado civil, ingreso familiar, religión, ciudad de residencia) y las relacionadas con la formación profesional (categoría profesional, tiempo de formación, tiempo de actuación en la enfermería y en las PICS, lugar de actuación en la enfermería y en las PICS, especialidad en la enfermería, conocimiento e interés en las PICS, formación en PICS).

### Tratamiento y análisis de los datos

 Los datos fueron exportados de REDCap directamente a una hoja de cálculo electrónica estructurada de *Microsoft Excel* , mediante procedimientos automáticos propios de la plataforma y analizados utilizando el programa estadístico *Statistical Package for the Social Sciences* (SPSS). 

Se realizó estadística descriptiva e inferencial. La caracterización de la muestra se llevó a cabo a partir de un análisis exploratorio mediante promedios, frecuencia y porcentaje. Para las comparaciones entre los participantes que realizaron y no realizaron formación en PICS se aplicó el test de Mann-Whitney. La distribución de los datos fue evaluada mediante el test de Shapiro-Wilk. Para evaluar las asociaciones entre la variable referente a la formación en PICS y las demás variables cualitativas se aplicó el test Chi-cuadrado de Pearson.

 Se construyó un modelo de regresión Poisson múltiple modificado jerárquico, con varianza robusta, considerando la formación en PICS como variable dependiente. En los resultados se presentaron las estimaciones obtenidas de razón de prevalencia, así como sus respectivos intervalos de confianza y p-valores. Para la realización de los análisis se utilizó el *software* estadístico SAS versión 9.4 y se consideró un nivel de significancia del 5%. 

### Aspectos éticos

 Se respetaron las normas y directrices éticas y el proyecto fue aprobado en el Comité de Ética en Investigación de la *Escola de Enfermagem de Ribeirão Preto* de la *Universidade de São Paulo* (EERP/USP) (CAAE 55755821.0.0000.5393). El proyecto está en conformidad con la Resolución 466/12. 

## Resultados

 De los 3.794 (100%) participantes, la mayoría eran mujeres (86,95%), con una edad promedio de 40,11 (DE 9,68) años, con 12,01 (DE 8,90) años de formación y 11,77 (DE 9,26) años de experiencia profesional; 479 profesionales tienen formación sobre PICS, lo que corresponde al 12,62% de los respondentes. La distribución de las variables sociodemográficas y de formación de los profesionales de enfermería que tienen o no formación en PICS, se presentan en la Tabla 1 . Las variables categóricas se expresan en números (porcentajes) y las variables continuas se expresan en media (desviación estándar). 

**Tabla 1 -:** Distribución de las variables sociodemográficas y de formación de los profesionales de enfermería con y sin formación en Prácticas Integrativas y Complementares en Salud en el estado de São Paulo (N* = 3794). São Paulo, Brasil, 2022

**Variables**	**No (3315)**		**Sí (479)**		**p-valor**
Edad	39,68	(9,57)	43,05	(9,92)	<0,0001 ^†^
Sexo					0,2177 ^‡^
Femenino	2874	87,12	425	12,88	
Masculino	441	89,09	54	10,91	
Raza/Color					< 0,0001 ^‡^
Amarillo/Blanco/Indígena	1965	85,47	334	14,53	
Moreno/Negro	1350	90,30	145	9,70	
Estado Civil					0,2013 ^‡^
Sin pareja	1044	88,40	137	11,60	
Con pareja	2271	86,91	342	13,09	
Ingreso familiar					< 0,0001 ^‡^
Hasta 2 salarios	770	94,59	44	5,41	
Entre 2 y 4 salarios	1293	91,18	125	8,82	
Entre 4 y 10 salarios	1053	81,75	235	18,25	
Por encima de 10 salarios	199	72,63	75	27,37	
Religión					0,0639 ^‡^
No	393	84,70	71	15,30	
Sí	2922	87,75	408	12,25	
Regiones					0,4090 ^‡^
São Paulo	2079	87,35	301	12,65	
Sorocaba/Campinas/ São José dos Campos	712	86,51	111	13,49	
Bauru/Marília/Presidente Prudente/Araçatuba	223	90,65	23	9,35	
São José do Rio Preto/ Ribeirão Preto/Araraquara	301	87,25	44	12,75	
Formación					< 0,0001 ^‡^
Técnico	1300	96,58	46	3,42	
Superior	760	92,57	61	7,43	
*Lato sensu*	1104	79,37	287	20,63	
*Stricto sensu*	151	63,98	85	36,02	
Formación profesional en enfermería (años)	11,48	8,62	15,72	9,85	< 0,0001 ^†^
Años de experiencia profesional en enfermería	11,20	9,01	15,73	9,98	< 0,0001 ^†^
Carga horaria semanal					< 0,0001 ^‡^
<= 40 horas	2341	85,56	395	14,44	
> 40 horas	974	92,06	84	7,94	
Vínculos laborales					0,7990 ^‡^
1	2164	87,12	320	12,88	
2 o más	502	88,07	68	11,93	
Sin vínculo	649	87,70	91	12,30	

Las variables categóricas se expresan en números (porcentajes) y las variables continuas se expresan en media (desviación estándar). No: Sin formación en Prácticas Integrativas y Complementarias en Salud (PICS); Sí: con formación en PICS.

* N= Número de participantes analizados

† Prueba de Mann-Whitney

‡ Pruebas chi-cuadrado

 La Tabla 2 muestra las características de la experiencia con PICS por parte de los profesionales de enfermería (n = 3794) con y sin formación en PICS. Se observa que el 93,95% de los profesionales con formación en PICS tuvieron alguna experiencia previa con las PICS, práctica o teórico-práctica, principalmente en la institución donde actúan. Esta experiencia estaba relacionada con la calidad de usuarios de los servicios de salud o como profesionales de la salud. Las variables categóricas se expresan en números (porcentajes) y las variables continuas se expresan en media (desviación estándar). 

**Tabla 2 -:** Características de la experiencia con Prácticas Integrativas y Complementarias en Salud (PICS) de profesionales de enfermería con y sin formación en PICS en el estado de São Paulo (N* = 3.794). São Paulo, Brasil, 2022

**Variables**	**No (3315)**		**Sí (479)**		**p-valor** ^†^
Informó saber qué son las PICS					< 0,0001
Sí	1842	79,64	471	20,36	
No	1473	99,46	8	0,54	
Vivencia o experiencia con las PICS					< 0,0001
Sí	988	68,71	450	31,29	
No	2327	98,77	29	1,23	
Experiencia					<0,0001
Teórica	375	87,01	56	12,99	
Práctica	453	70,67	188	29,33	
Teórico-Práctica	160	43,72	206	56,28	
No posee experiencia	2327	98,77	29	1,23	
Rol en esta primera experiencia:					<0,0001
Usuario/a (uso propio)	230	65,71	120	34,29	
Acompañante de família, amigos	79	91,86	7	8,14	
Estudiante (durante su formación en enfermería)	248	81,05	58	18,95	
Estudiante (otra formación que no es enfermería)	27	21,95	96	78,05	
Profesional de salud (contacto en el institución donde actúa)	404	70,51	169	29,49	
No posee experiencia	2327	98,77	29	1,23	

Las variables categóricas se expresan en números (porcentajes) y las variables continuas se expresan en media (desviación estándar). No: Sin formación en PICS; Sí: Con formación en PICS.

* N= Número de participantes analizados

† p= Nivel de significancia analizado por el Teste chi-cuadrado

 A partir de las características de la experiencia con PICS por profesionales de enfermería presentadas, se construyó un modelo del perfil de los profesionales de enfermería, con formación en PICS ( Tabla 3 ). Las variables edad, no tener religión formal, formación superior y especialización ( *lato* y/o *stricto sensu* ), trabajar en una carga horaria igual o menor que 40 horas semanales, informar qué son PICS presentaron significancia estadística. 

 Las características de la formación en PICS por profesionales de enfermería en el estado de São Paulo se presentan en la Figura 1 . Las prácticas más identificadas fueron la auriculoterapia, la Medicina Tradicional China/acupuntura y el *reiki* . En cuanto a las características de los cursos realizados para la formación, prevalecieron las modalidades de curso libre (educación profesional con carga horaria reducida, como talleres y *workshops* ), presenciales y teórico-prácticos. 

 La carga horaria representa el número de horas dedicadas a la formación teórico-práctica en cada curso, ofreciendo una visión general del tiempo necesario invertido para adquirir conocimientos y habilidades en cada uno de los enfoques terapéuticos. Destacan la acupuntura, la naturopatía y la terapia comunitaria como aquellas con mayor carga horaria media de formación, y la biodanza, la arteterapia y la osteopatía, con menor carga horaria. Los valores de medias presentados en la Tabla 4 referentes a la carga horaria de los cursos en PICS mostraron una desviación estándar significativamente alta, indicando que hay una variación considerable en la carga horaria de los cursos ofrecidos, incluso dentro de una misma modalidad de PICS. 

**Tabla 3 -:** Perfil de los profesionales de enfermería con formación en Prácticas Integrativas y Complementarias en Salud en el estado de São Paulo (N* = 3.794). São Paulo, Brasil, 2022

**Variables**	**Paso 1**				**Paso 2**				**Paso 3**			
	**RP** ^†^	**I.C.** ^‡^ **(95%)**		**p-valor** ^¶^	**RP** ^†^	**I.C.** ^‡^ **(95%)**		**p-valor** ^¶^	**RP** ^†^	**I.C.** ^‡^ **(95%)**		**p-valor** ^¶^
		**L.I.** ^§^	**L.S.** ^[Table-fn tfn08]||^			**L.I.** ^§^	**L.S.** ^[Table-fn tfn08]||^			**L.I.** ^§^	**L.S.** ^[Table-fn tfn08]||^	
Edad	1,02	1,01	1,03	**< 0,0001**	1,02	1,01	1,02	**0,0002**	1,01	1,01	1,02	**0,0004**
Sexo femenino	1,23	0,95	1,60	0,1219								
Raza/Color (Amarillo/Blanco/Indígena)	1,21	1,01	1,46	**0,0389**	1,10	0,92	1,32	0,3036				
Ingreso familiar (Entre 2 y 4 salarios**)	1,56	1,12	2,18	**0,0084**	1,08	0,78	1,51	0,6378	1,09	0,79	1,49	0,6048
Ingreso familiar (Entre 4 y 10 salarios**)	3,02	2,21	4,13	**< 0,0001**	1,30	0,94	1,79	0,1129	1,15	0,84	1,58	0,3680
Ingreso familiar (Por encima de 10 salarios**)	4,16	2,90	5,96	**< 0,0001**	1,44	1,00	2,08	0,0505	1,16	0,82	1,63	0,4105
Religión (No)	1,27	1,01	1,60	**0,0373**	1,25	1,01	1,56	**0,0416**	1,23	1,02	1,48	**0,0342**
Formación (Superior)					1,97	1,35	2,88	**0,0004**	1,46	1,02	2,09	**0,0404**
Formación ( *Lato sensu* )					4,88	3,54	6,71	**< 0,0001**	2,77	2,03	3,78	**< 0,0001**
Formación ( *Stricto sensu* )					7,39	5,16	10,58	**< 0,0001**	3,32	2,36	4,66	**< 0,0001**
Carga horaria semanal (<= 40 horas)					1,57	1,26	1,96	**< 0,0001**	1,40	1,14	1,71	**0,0013**
Informó saber qué son PICS ^††^ (Sí)									4,84	2,35	10,01	**< 0,0001**
Vivencia o experiencia con las PICS ^††^ (Sí)									10,47	7,18	15,28	**< 0,0001**

* N = Número de participantes analizados

† RP = Razón de prevalencia. Se estimó la probabilidad de presentar el resultado “Sí”

‡ I.C. = Intervalo de confianza

§ L.I. = Límite inferior

|| L.S. = Límite superior

¶ p-valor = Nivel de significancia a partir del Modelo de Regresión de Poisson múltiple modificado jerárquico, con varianza robusta

** Salario mínimo vigente = R$ 1.212,00, Brasil, 2022

†† PICS = Prácticas Integrativas y Complementarias en Salud

**Figura 1:** - Características autoinformadas de los cursos de formación en Prácticas Integrativas y Complementarias en Salud realizados por profesionales de enfermería en el estado de São Paulo (N* = 479). São Paulo, Brasil, 2022

*N = Número de participantes analizados; ^†^ PICS = Prácticas Integrativas y Complementarias en Salud; ^‡^ EaD = Educación a distancia; ^§^ T/P = Teórico/Práctico

**Tabla 4 -:** Carga horaria autoinformada de los cursos de formación en Prácticas Integrativas y Complementarias en Salud realizados por profesionales de enfermería en el Estado de São Paulo (N* = 479). São Paulo, Brasil, 2022

**PICS**	N*	**Promedio**	**Desviación-estándar**	**Mínimo**	**Q1** ^†^	**Mediana**	**Q3** ^‡^	**Máximo**
Antroposofía	7	167,14	180,44	30,00	40,00	120,00	220,00	540,00
Apiterapia	1	56,00	-	-	-	-	-	-
Aromaterapia	56	159,80	273,83	5,00	20,00	40,00	180,00	1744,00
Arteterapia	1	30,00	-	-	-	-	-	-
Auriculoterapia	165	124,45	211,10	0,00	30,00	75,00	100,00	1300,00
Ayurveda	6	298,67	401,93	12,00	60,00	200,00	220,00	1100,00
Biodanza	3	10,00	8,66	5,00	5,00	5,00	20,00	20,00
Constelación	27	232,22	223,69	0,00	80,00	150,00	360,00	900,00
Cromoterapia	22	59,64	77,67	2,00	30,00	40,00	60,00	360,00
Danza Circular	7	94,00	137,77	16,00	20,00	40,00	90,00	400,00
Geoterapia	0	-	-	-	-	-	-	-
Homeopatía	6	400,00	301,70	42,00	58,00	450,00	600,00	800,00
Hipnoterapia	5	42,00	30,92	0,00	26,00	40,00	72,00	72,00
Imposición de manos	7	64,57	44,22	20,00	24,00	50,00	120,00	120,00
MTC/Acupuntura	125	860,59	584,22	3,00	380,00	800,00	1200,00	3500,00
Meditación	24	94,54	86,94	2,00	30,00	50,00	170,00	300,00
Musicoterapia	4	35,00	32,72	4,00	12,00	28,00	58,00	80,00
Naturopatía	13	748,46	777,49	30,00	460,00	480,00	480,00	3000,00
Osteopatía	1	30,00	-	-	-	-	-	-
Ozonioterapia	31	218,32	382,79	20,00	120,00	120,00	200,00	2200,00
Quiropraxia	1	40,00	-	-	-	-	-	-
Plantas medicinales y fito	17	221,29	214,61	8,00	60,00	120,00	360,00	720,00
Reflexoterapia	19	252,89	458,21	8,00	40,00	100,00	288,00	2000,00
*Reiki*	93	125,37	394,17	1,00	16,00	30,00	60,00	2400,00
*Shantala*	8	39,25	58,38	8,00	8,00	19,00	36,00	180,00
Terapia Comunitaria	10	409,80	583,06	8,00	30,00	210,00	500,00	1940,00
Terapia Floral	37	191,73	234,68	4,00	30,00	90,00	360,00	1200,00
Termalismo/Crenoterapia	0	-	-	-	-	-	-	-
Yoga	7	259,14	212,88	14,00	120,00	180,00	500,00	600,00
Otras	74	398,42	655,09	0,00	40,00	135,00	360,00	4138,00

* N = Número de participantes analizados

† Q1 = Primer cuartil

‡ Q3 = Tercer cuartil

## Discusión

La población con formación en PICS presentó una edad promedio de 43,05 años, con predominio del sexo femenino, raza/color blanco, amarillo e indígena, que viven con parejas, sin declaración de religión, ingresos entre 4 y 10 salarios mínimos, con formación superior especializada y, en promedio, 15 años de formación y experiencia profesional. Se observó una diferencia significativa en cuanto a la edad promedio y al tiempo de formación y experiencia profesional, siendo mayor entre la población con formación en PICS.

 En cuanto al conocimiento, el 39% (n=1481) informó desconocimiento sobre PICS. Resultado similar fue identificado entre enfermeras iraníes, cuyo conocimiento sobre PICS fue evaluado como bajo ^(^ 17 ^)^ . Un estudio de mapeo que trabajó con una muestra de conveniencia con retorno de diez países europeos no encontró un enfoque consistente en la formación de enfermeros en PICS, pues aunque se imparten cursos en institutos regulares de enseñanza, no están integrados a la enseñanza regular de grado ^(^ 18 ^)^ . Aunque los hallazgos de este estudio son convergentes con el estudio mencionado, la interpretación de los datos debe considerar las características histórico-culturales de los distintos países respecto a las racionalidades médicas y sistemas tradicionales de curación, así como las características de las políticas de formación y la actuación de los profesionales en PICS. 

 En el presente estudio, los profesionales con formación en PICS mencionaron que las prácticas más frecuentes fueron la auriculoterapia, la Medicina Tradicional China/acupuntura y el *reiki* . Este resultado difiere de los datos de la Encuesta Nacional de Salud, que muestra acupuntura, homeopatía, plantas medicinales, meditación y yoga ^(^ 8 ^)^ . La auriculoterapia como la PICS más frecuente en la población estudiada puede estar relacionada con la capacitación que se ha ofrecido en Brasil, desde 2016, para profesionales de enseñanza superior que actúan en la APS. En estos cursos, los profesionales participantes son del sexo femenino, profesionales de enfermería con una edad promedio de 36,8 años ^(^ 19 ^)^ . Este perfil se asemeja a los profesionales de enfermería del estado de São Paulo investigados en el presente estudio. 

 En comparación con países europeos, se observó que las principales PICS ofrecidas para la formación en enfermería son masajes, meditación, mindfulness y técnicas de relajación ^(^ 18 ^)^ . La formación, tanto a nivel nacional como mundial, representa una brecha que merece esfuerzos para ampliar el acceso a la capacitación de los profesionales de la salud, especialmente de enfermería, atendiendo a resultados de investigaciones internacionales y nacionales que indican la falta de conocimiento como una de las principales debilidades para la ampliación de las ofertas de PICS ^(^ 17 ^,^ 20 ^)^ . Este hallazgo pone en perspectiva la discusión sobre la formación inicial y continua de los profesionales de Enfermería en lo que respecta a PICS, ya que se observa una falta de enfoque consistente tanto en Brasil como en Europa en la educación de los enfermeros en PICS y enfermería integrativa ^(^ 18 ^)^ . Para la enfermería europea, al igual que la brasileña, se imparten cursos. Sin embargo, no existe una directriz nacional que integre las PICS al currículo regular de enseñanza ^(^ 18 ^)^ . 

 Se constató, en el presente estudio, que los profesionales con alguna formación en PICS también tuvieron contacto previo con alguna práctica, ya sea durante su formación como profesional o en la institución en la que actúan. Así, se destaca la importancia de que los cursos formativos ofrezcan disciplinas en sus currículos para sensibilizar a los futuros profesionales sobre la temática y presentar las PICS como un enfoque de cuidado a la salud. En Brasil, se observa que las graduaciones en Enfermería, Medicina y Odontología tienen como característica la oferta de disciplinas sobre PICS electivas, teóricas y no integradas a las matrices curriculares ^(^ 21 ^-^ 22 ^)^ . 

 En relación con la formación, los hallazgos reflejan la variabilidad en la carga horaria para cada una de las diferentes formaciones. Este resultado corrobora la ausencia de directrices regulatorias respecto a un currículo mínimo, con consecuentes discrepancias. En este sentido, urge la necesidad de establecer resoluciones que orienten a la categoría sobre la carga horaria mínima para cada capacitación para cada práctica. Los resultados muestran que los profesionales comprendieron como “formación” tanto cursos libres, de difusión, conferencias, talleres, como cursos de capacitación, demostrando ausencia de claridad sobre lo que representa un proceso formativo. La variabilidad en la formación es un aspecto preocupante señalado en la literatura, constituyéndose en una debilidad para la propia área de las PICS, para la formación profesional y para los usuarios de los servicios de salud ^(^ 17 ^)^ . 

 Desde la formación hasta la actuación también hay desafíos relativos a la disputa por espacio entre categorías profesionales, a la cultura institucional, a la disponibilidad de tiempo y de recursos para la oferta de las PICS ^(^ 23 ^)^ . Estas repercusiones se observan en contratos laborales en Enfermería que frecuentemente no abarcan o reconocen la oferta de las PICS como parte de sus atribuciones profesionales. También hay fragilidades en la gestión de los servicios de salud, lo que dificulta la comprensión, el reconocimiento y la oferta de PICS, materializada en la falta de disponibilidad de recursos materiales y en la divulgación sistematizada. Esta problemática se refleja directamente en la discrepancia entre la oferta real y la registrada en los sistemas de información, evidenciando la problemática del registro subestimado de las PICS ^(^ 24 ^)^ . 

 Por otro lado, la formación en PICS y la oferta entre los servicios han estado en aumento en el territorio nacional ^(^ 8 ^,^ 21 ^-^ 22 ^)^ . Esta situación permite identificar los motivos por los cuales los profesionales de Enfermería - la mayor fuerza laboral del Sistema Único de Salud - persisten en la inversión para la formación y oferta de PICS en los servicios de salud. Esta realidad convoca a las entidades gubernamentales y organismos profesionales a analizar el marco ético-legal y técnico que sostiene las acciones. 

 En cuanto a la calificación, se constató en la población investigada que hubo búsqueda de calificaciones en las 29 terapias complementarias dispuestas en la PNPIC ^(^ 4 ^)^ , sin embargo, en las Resoluciones específicas de Enfermería, solo diez prácticas están incluidas como especialidades de la profesión ^(^ 7 ^)^ . La dimensión institucional se incluye en esta problemática, como la elaboración y disponibilidad de Protocolos Clínicos y Procedimientos Operacionales Estándar que posibilitan la actuación de Enfermería en la oferta de PICS en los servicios de salud. 

 Se destaca que las PICS han ganado protagonismo en el escenario mundial. En 2023 se llevó a cabo la primera cumbre Global de Medicina Tradicional, en la India, paralelamente a la reunión interministerial de salud compuesta por los miembros del Grupo de los 20 (G20). El objetivo de la cumbre fue registrar el compromiso político de los estados miembros en ofrecer a sus poblaciones la Medicina Tradicional basada en evidencias para atender las necesidades de salud y bienestar ^(^ 25 ^)^ . Al tratarse de una temática universal de salud priorizada por una entidad global, corresponde a Enfermería incorporar tales prácticas en el ejercicio de su profesión. 

Este estudio tiene limitaciones que se refieren principalmente a los datos obtenidos en relación con la formación. Se observó una desviación estándar considerable en la carga horaria de formación entre los encuestados del estudio, indicando que posiblemente conferencias fueron consideradas por los profesionales como actividad de carácter formativo. En este sentido, se sugiere que en futuros estudios la pregunta investigativa que oriente tal cuestión esté vinculada a la práctica ejercida en PICS, como por ejemplo, “¿Cuántas horas se invirtieron en su formación que le calificaron para aplicar esta PICS?”.

Las contribuciones del estudio se refieren al levantamiento de los aspectos relacionados con la formación y la actuación de Enfermería en la unidad federativa del país que tiene la mayor concentración de profesionales, respondiendo a un vacío científico internacional y nacional sobre el perfil sociodemográfico y profesional de Enfermería en PICS. Basado en los hallazgos, se argumenta sobre la importancia de políticas públicas en el área de la salud y su articulación e información a las políticas de formación de recursos humanos.

## Conclusión

En el estado de São Paulo, aunque hay un contingente considerable de profesionales de enfermería que tienen interés por las PICS, casi la mitad de los profesionales desconocen tales prácticas. La formación de los profesionales en el área se da, principalmente, entre enfermeras de alrededor de cuarenta años de edad, con especialización y, aproximadamente, quince años de experiencia profesional.

Se destaca que las directrices mínimas en relación con la formación deben ser consideradas para la capacitación de los profesionales de Enfermería en PICS, así como en las demás profesiones de la salud. La estandarización es fundamental para la continuidad y ampliación del acceso de la población a las PICS, con garantía de profesionales debidamente cualificados para la oferta segura de los recursos terapéuticos en los diversos escenarios institucionales.

# Translation: Perfil sociodemográfico e de formação dos profissionais de Enfermagem do estado de São Paulo em relação às Práticas Integrativas e Complementares em Saúde

**DOI:** 10.1590/1518-8345.7144.4204

**Published:** 2024-09-23

**Authors:** Juliana Rizzo Gnatta, Thiago da Silva Domingos, Edilaine Cristina da Silva Gherardi-Donato, Suzimar de Fátima Benato Fusco, Leonice Fumiko Sato Kurebayashi, Talita Pavarini Borges

**Affiliations:** 1 Universidade de São Paulo, Escola de Enfermagem, Departamento Médico-Cirúrgico, São Paulo, SP, Brasil; 2 Brasilian Ctr Evidence Based Healthcare, JBI Ctr Excellence JBI Brasil, Sao Paulo, SP, Brasil; 3 Universidade Federal de São Paulo, Escola Paulista de Enfermagem, Departamento de Enfermagem Clínica e Cirúrgica, São Paulo, SP, Brasil; 4 Universidade de São Paulo, Escola de Enfermagem de Ribeirão Preto, Departamento de Enfermagem Psiquiátrica e Ciências Humanas, Centro Colaborador de la OPS/OMS para el Desarrollo de la Investigación en Enfermería, Ribeirão Preto, SP, Brasil; 5 Universidade Estadual de Campinas, Faculdade de Enfermagem, Departamento de Enfermagem Médico-Cirúrgica, Campinas, SP, Brasil; 6 Associação Brasileira de Enfermeiros de Centro Cirúrgico, Recuperação Anestésica e Centro de Material e Esterilização, Comitê de Ensino e Pesquisa em Enfermagem Perioperatória, São Paulo, SP, Brasil; 7 Universidade de São Paulo, Escola de Enfermagem, São Paulo, SP, Brasil; 8 Instituto de Terapia Integrada e Oriental, São Paulo, SP, Brasil; 9 Conselho Regional de Enfermagem de São Paulo, Grupo de Trabalho em Práticas Integrativas e Complementares em Saúde, São Paulo, SP, Brasil; 10 Instituto Pavarini, São Paulo, SP, Brasil

**Keywords:** Enfermagem, Papel do Profissional de Enfermagem, Formação Profissional, Perfil Profissional, Terapias Complementares, Métodos Terapêuticos Complementares

## Abstract

**Objetivo:**

analisar o perfil sociodemográfico e de formação dos profissionais de Enfermagem do estado de São Paulo em relação às Práticas Integrativas e Complementares em Saúde.

**Método:**

estudo transversal, desenvolvido com 3.794 profissionais de Enfermagem do estado de São Paulo, provenientes de 645 municípios. Para a coleta de dados, utilizou-se um questionário *online* contendo as variáveis sociodemográficas e de formação profissional. Foi construído um modelo de regressão de Poisson múltiplo hierárquico, considerando a formação em práticas como variável dependente, com nível de significância de 5%.

**Resultados:**

dos 3.794 (100%) participantes, 479 (12,62%) tinham formação em Práticas Integrativas e Complementares em Saúde. As práticas mais frequentes foram a auriculoterapia, a Medicina Tradicional Chinesa/acupuntura e o *Reiki* . As modalidades de formação predominantes foram cursos livres, presenciais e teórico-práticos. As variáveis idade, sem religião formal, nível superior de formação e especialização, carga horária de trabalho e conhecimento e experiência prévia sobre práticas integrativas apresentaram significância.

**Conclusão:**

os resultados indicam a necessidade de incentivar e expandir a formação em Práticas Integrativas e Complementares em Saúde dos profissionais de Enfermagem, a fim de fortalecer sua atuação nos serviços de saúde.

## Destaques

**(1)** 39% dos profissionais de Enfermagem desconhecem as PICS.

**(2)** Baixa proporção (14,5%) de profissionais de Enfermagem com formação em PICS.

**(3)** Auriculoterapia, MTC/acupuntura e *Reiki* são as formações mais frequentes.

**(4)** Cursos livres, presenciais e teórico-práticos são predominantes na formação.

**(5)** Há grande discrepância na carga horária de formação em PICS.

## Introdução

 As Práticas Integrativas e Complementares em Saúde (PICS) são recursos terapêuticos empregados de forma complementar ao tratamento convencional, isoladamente ou como parte das racionalidades médicas, que são sistemas médicos complexos ^(^ 1 ^)^ compostos por conhecimentos e práticas integradas e estruturadas. Possuem caráter interdisciplinar, com foco na promoção da saúde e prevenção de doenças crônicas a partir de mudanças no estilo de vida e autocuidado ^(^ 1 ^)^ . As PICS caminham na direção de um novo paradigma, cujo enfoque principal é a saúde, por meio da busca de equilíbrio do indivíduo em relação ao meio natural e social a que pertence. 

 Em 2013, a Organização Mundial de Saúde (OMS) ^(^ 2 ^)^ adotou no documento de Estratégia da OMS sobre medicina tradicional 2014-2023, que foi prorrogado até 2025 ^(^ 2 ^)^ , a denominação Medicinas Tradicionais, Complementares e Integrativas (MTCI) para as modalidades que abrangem (1) racionalidades médicas, como Medicina Tradicional Chinesa, Ayurveda indiana, homeopatia e Medicina Antroposófica, entre outras; e (2) práticas terapêuticas, as quais incluem as intervenções mente e corpo, terapias de manipulação corporal, naturais e energéticas ^(^ 1 ^,^ 3 ^)^ . As diferentes práticas terapêuticas foram denominadas no Brasil como PICS, com a aprovação, pelo Ministério da Saúde, da Política Nacional de Práticas Integrativas e Complementares (PNPIC) no Sistema Único de Saúde (SUS) no ano de 2006 ^(^ 4 ^)^ . 

 Em 2018, o conjunto de práticas alcançou 29 modalidades terapêuticas reconhecidas pelo Ministério da Saúde e oferecidas pelo SUS ^(^ 5 ^)^ . Os princípios que regem as PICS são congruentes com os da Enfermagem, pois partem de uma visão centrada no ser humano para disponibilizar uma estrutura de cuidados integrais que possa ser oferecida em todos os ambientes clínicos, a fim de promover a saúde e o bem-estar, sendo este conceito denominado Enfermagem integrativa ^(^ 6 ^)^ . 

 As Terapias Holísticas e Complementares foram afirmadas como especialidade de Enfermagem por meio da Resolução do Conselho Federal de Enfermagem (Cofen) nº 581 de 2018 ^(^ 7 ^)^ , assegurando o respaldo para a atuação do profissional de Enfermagem nesse cenário, bem como para desenvolver pesquisas na área das PICS. 

 Observa-se uma crescente utilização das PICS no mundo ^(^ 6 ^)^ e no Brasil durante a última década, atingindo 5,2% da população maior de 18 anos ^(^ 8 ^)^ . Nas regiões Norte e Sul há uma predominância na população caracterizada pelo sexo feminino, cor/raça branca, com maior renda, escolaridade e faixa etária. Acupuntura, homeopatia, plantas medicinais, meditação e yoga são as PICS de maior prevalência no país ^(^ 8 ^)^ . 

 Numericamente, a Enfermagem é uma categoria que tem representatividade nacional dentre as profissões da saúde, contando com aproximadamente 2.873.087 de profissionais entre enfermeiros, técnicos de Enfermagem, auxiliares e obstetrizes. O estado de São Paulo tem 735.296 profissionais ^(^ 9 ^)^ , representando cerca de 26% do contingente da Enfermagem brasileira. 

 Um longo caminho ainda precisa ser percorrido para fortalecer a utilização das PICS pelos enfermeiros. O Cofen tem tido um papel fundamental na defesa das PICS no SUS, no sentido de regulamentar, fortalecer, estimular e difundir as diferentes práticas para benefício de usuários, trazendo maior autonomia e recursos terapêuticos para a Enfermagem ^(^ 10 ^-^ 12 ^)^ . 

 Com relação a São Paulo, as “Câmaras Técnicas” do Conselho Regional de Enfermagem do Estado de São Paulo (Coren-SP) instituíram um Grupo de Trabalho PICS, para estudar a regulamentação da atuação do enfermeiro nesta área, fazer um levantamento daqueles que já trabalham nela e subsidiar novas propostas, fundamentando pareceres sobre as práticas para a categoria ^(^ 13 ^)^ . 

 A oferta de PICS na Atenção Primária à Saúde (APS) pela Enfermagem no estado de São Paulo foi recuperada em artigo publicado em 2022, evidenciando o registro crescente desses procedimentos que, entretanto, teve um declínio importante durante a pandemia da COVID-19 ^(^ 14 ^)^ . Os registros da aplicação de PICS por enfermeiros nos sistemas de informação em saúde oficiais (DATASUS) confirmam a atuação da Enfermagem, não havendo dados na literatura científica que orientem quanto ao perfil e ao preparo desses profissionais para os procedimentos realizados. 

 Diferentes formatos de capacitações em PICS têm sido oferecidos para os profissionais. Cursos gratuitos são disponibilizados pelo Ministério da Saúde em parceria com universidades para os profissionais de nível superior que atuam na APS ^(^ 15 ^)^ . Além destes, instituições públicas e privadas oferecem cursos gratuitos e pagos para capacitação nas diferentes PICS, não havendo um mapeamento oficial que congregue estas iniciativas. 

Diante do exposto, o presente estudo buscou preencher uma lacuna de conhecimento sobre como tem se dado a inserção das PICS realizadas especialmente pela Enfermagem brasileira e nos diferentes estados, seja no âmbito assistencial ou formativo. Dessa forma, teve como objetivo analisar o perfil sociodemográfico e de formação dos profissionais de Enfermagem do estado de São Paulo em relação às Práticas Integrativas e Complementares em Saúde.

## Método

### Tipo do estudo

 Estudo observacional, transversal e descritivo, norteado pela diretriz STROBE - *Strengthening the Reporting of Observational Studies in Epidemiology(* ^(^ 16 ^)^ . 

### Cenário

A pesquisa abrangeu os profissionais de Enfermagem do estado de São Paulo que possuíam registro de inscrição ativa no Coren-SP durante o período do estudo.

### Período

A pesquisa foi realizada no período de 27 de julho a 17 de setembro de 2022.

### População

A população foi constituída por profissionais de Enfermagem (auxiliares, técnicos, enfermeiros e obstetrizes) que atuam no estado de São Paulo.

### Critérios de seleção

Foram considerados como critérios de inclusão: profissional de Enfermagem ativo registrado no Coren-SP, com idade mínima de 18 anos e acesso à internet, de ambos os sexos. Foram excluídos da amostra aqueles profissionais que não responderam a todas as questões do questionário de coleta de dados.

### Definição da amostra

Cálculo do tamanho amostral com universo e prevalência desconhecidos (50%) e erros padronizados (2% alfa e 20% beta), sem pressupor erros e falhas, resultando em amostra esperada de, no mínimo, 2396 voluntários.

### Recrutamento

 O recrutamento dos profissionais de Enfermagem ocorreu de forma *online* . Foi realizada uma ampla divulgação da pesquisa, nos meios virtuais mais utilizados pela população-alvo, sendo postados *links* do questionário em diversos ambientes virtuais abertos como: *site* de Universidades, *site* do Coren-SP, redes sociais ( *Whatsapp* , *Instagram* e *Facebook* ) e *e-mails* , com possibilidade de compartilhamento para ampliar o alcance. Adicionalmente, todos os profissionais com licença profissional ativa no Coren-SP receberam o convite para participar do estudo por meio de correio eletrônico do próprio Conselho. No corpo do *e-mail* foi enviada mensagem de convite com as informações sobre a pesquisa e a participação, finalizada com um *link* que direcionava o profissional ao Termo de Consentimento Livre e Esclarecido (TCLE) eletrônico. Nessa página, após lerem as explicações sobre a pesquisa e sobre os aspectos éticos, aqueles que aceitaram participar expressaram a sua concordância pelo TCLE eletrônico. 

Desta forma, iniciaram a pesquisa 6208 pessoas, sendo que 77 se recusaram a participar e 2337 não preencheram o questionário completamente, representando 38,88% de perdas. Portanto, a amostra foi constituída por 3.794 profissionais de Enfermagem de 645 municípios do estado de São Paulo.

### Coleta de dados

 Para a coleta de dados foi utilizado um questionário *online* autoaplicável, desenvolvido pelos autores do estudo (material suplementar). A coleta de dados ocorreu por meio da ferramenta de pesquisa *Research Electronic Data Capture* (REDCap), com a finalidade de auxiliar o processo de coleta e gerenciamento dos dados do estudo. 

 O questionário foi construído no REDCap para garantir que se considerasse apenas uma resposta por participante na análise dos dados, solicitando-se o *e-mail* do participante no ato do preenchimento. Apenas uma resposta com *e-mails* duplicados foi validada. 

### Variáveis do estudo

As variáveis de interesse para alcance dos objetivos do estudo foram as variáveis demográficas (idade, sexo, raça/cor, estado civil, renda familiar, religião, cidade onde reside) e as relacionadas à formação profissional (categoria profissional, tempo de formação, tempo de atuação na Enfermagem e nas PICS, local de atuação na Enfermagem e nas PICS, especialidade na Enfermagem, conhecimento e interesse nas PICS, formação em PICS).

### Tratamento e análise dos dados

 Os dados foram exportados do REDCap diretamente para uma planilha eletrônica estruturada do Microsoft Excel, por procedimentos automáticos próprios da plataforma, e analisados utilizando-se o programa estatístico *Statistical Package for the Social Sciences* (SPSS). 

Foi realizada estatística descritiva e inferencial. A caracterização da amostra foi feita a partir de análise exploratória por meio de médias, frequência e porcentagem. Para as comparações entre os participantes que realizaram e não realizaram formação em PICS foi aplicado o teste de Mann-Whitney. A distribuição dos dados foi avaliada por meio do teste de Shapiro-Wilk. Para avaliar as associações entre a variável referente à formação em PICS e as demais variáveis qualitativas foi aplicado o teste Qui-quadrado de Pearson.

 Foi construído um modelo de regressão Poisson múltiplo modificado hierárquico, com variância robusta, considerando a formação em PICS como variável dependente. Nos resultados foram apresentadas as estimativas obtidas de razão de prevalência, assim como os seus respectivos intervalos de confiança e p-valores. Para a realização das análises foi utilizado o *software* estatístico SAS versão 9.4 e considerado um nível de significância de 5%. 

### Aspectos éticos

As normas e as diretrizes éticas foram respeitadas e o projeto foi aprovado no Comitê de Ética em Pesquisa da Escola de Enfermagem de Ribeirão Preto da Universidade de São Paulo (EERP/USP) (CAAE 55755821.0.0000.5393). O projeto está em conformidade com a Resolução 466/12.

## Resultados

 Dos 3.794 (100%) participantes, a maioria era de mulheres (86,95%), com média de idade de 40,11 (DP 9,68) anos, 12,01 (DP 8,90) anos de formação e 11,77 (DP 9,26) anos de experiência profissional; 479 profissionais têm formação sobre PICS, o que corresponde a 12,62% dos respondentes. A distribuição das variáveis sociodemográficas e de formação dos profissionais de Enfermagem que têm ou não formação em PICS estão apresentadas na Tabela 1 . As variáveis categóricas estão expressas em números (percentuais) e as variáveis contínuas estão expressas em média (desvio-padrão). 

 A Tabela 2 mostra as características da experiência com PICS por profissionais de Enfermagem (n = 3794) com e sem formação em PICS. Observa-se que 93,95% dos profissionais com formação em PICS tiveram alguma experiência prévia com as PICS, prática ou teórico-prática, principalmente na instituição onde atuam. Essa experiência estava relacionada na qualidade de usuários dos serviços de saúde ou como profissionais de saúde. As variáveis categóricas estão expressas em números (percentuais) e as variáveis contínuas estão expressas em média (desvio-padrão). 

**Tabela 1 -:** Distribuição das variáveis sociodemográficas e de formação dos profissionais de Enfermagem com e sem formação em Práticas Integrativas e Complementares em Saúde no estado de São Paulo (N* = 3794). São Paulo, Brasil, 2022

**Variáveis**	**Não (3315)**		**Sim (479)**		**p-valor**
Idade	39,68	(9,57)	43,05	(9,92)	< 0,0001 ^†^
Sexo					0,2177 ^‡^
Feminino	2874	87,12	425	12,88	
Masculino	441	89,09	54	10,91	
Raça/Cor					0,0001 ^‡^
Amarelo/Branca/Indígena	1965	85,47	334	14,53	
Parda/Preta	1350	90,30	145	9,70	
Estado Civil					0,2013 ^‡^
Sem parceiro	1044	88,40	137	11,60	
Com parceiro	2271	86,91	342	13,09	
Renda familiar					0,0001 ^‡^
Até 2 salários	770	94,59	44	5,41	
Entre 2 e 4 salários	1293	91,18	125	8,82	
Entre 4 e 10 salários	1053	81,75	235	18,25	
Acima de 10 salários	199	72,63	75	27,37	
Religião					0,0639 ^‡^
Não	393	84,70	71	15,30	
Sim	2922	87,75	408	12,25	
Regiões					0,4090 ^‡^
São Paulo	2079	87,35	301	12,65	
Sorocaba/Campinas/São José dos Campos	712	86,51	111	13,49	
Bauru/Marília/Presidente Prudente/Araçatuba	223	90,65	23	9,35	
São José do Rio Preto/Ribeirão Preto/Araraquara		301	87,25	44	12,75
Formação					0,0001 ^‡^
Técnico	1300	96,58	46	3,42	
Superior	760	92,57	61	7,43	
*Lato sensu*	1104	79,37	287	20,63	
*Stricto sensu*	151	63,98	85	36,02	
Formação profissional na Enfermagem (anos)	11,48	8,62	15,72	9,85	0,0001 ^†^
Anos de experiência profissional na Enfermagem	11,20	9,01	15,73	9,98	0,0001 ^†^
Carga horária semanal					0,0001 ^‡^
= 40 horas	2341	85,56	395	14,44	
40 horas	974	92,06	84	7,94	
Vínculos empregatícios					0,7990 ^‡^
1	2164	87,12	320	12,88	
2 ou mais	502	88,07	68	11,93	
Sem vínculo	649	87,70	91	12,30	

As variáveis categóricas estão expressas em números (percentuais) e as variáveis contínuas estão expressas em média (desvio-padrão). Não: Sem formação em Práticas Integrativas e Complementares em Saúde (PICS); Sim: com formação em PICS. *N = Número de participantes analisados

† Teste de Mann-Whitney

‡ Teste Qui-quadrado

**Tabela 2 -:** Características da experiência com Práticas Integrativas e Complementares em Saúde (PICS) de profissionais de Enfermagem com e sem formação em PICS no estado de São Paulo (N* = 3.794). São Paulo, Brasil, 2022

**Variáveis**	**Não (3315)**		**Sim (479)**		**p-valor** ^†^
Informou saber o que são PICS					0,0001
Sim	1842	79,64	471	20,36	
Não	1473	99,46	8	0,54	
Vivência ou experiência com as PICS					0,0001
Sim	988	68,71	450	31,29	
Não	2327	98,77	29	1,23	
Experiência					0,0001
Teórica	375	87,01	56	12,99	
Prática	453	70,67	188	29,33	
Teórico-Prática	160	43,72	206	56,28	
Não possui experiência	2327	98,77	29	1,23	
Papel nesta primeira experiência:					0,0001
Usuário/a (uso próprio)	230	65,71	120	34,29	
Acompanhante de família, amigos	79	91,86	7	8,14	
Estudante (durante sua formação na Enfermagem)	248	81,05	58	18,95	
Estudante (outra formação que não Enfermagem)	27	21,95	96	78,05	
Profissional de saúde (contato na instituição onde atua)	404	70,51	169	29,49	
Não possui experiência	2327	98,77	29	1,23	

As variáveis categóricas estão expressas em números (percentuais) e as variáveis contínuas estão expressas em média (desvio-padrão). Não: Sem formação em PICS; Sim: Com formação em PICS. *N = Número de participante analisados

† p = Nível de significância analisado pelo Teste Qui-quadrado

 A partir das características da experiência com PICS de profissionais de Enfermagem apresentadas, construiu-se um modelo do perfil dos profissionais de Enfermagem com formação em PICS ( Tabela 3 ). As variáveis idade, não ter religião formal, formação superior e especialização ( *lato* e/ou *stricto sensu* ), trabalhar em carga horária igual ou menor que 40 horas semanais, e informar o que são PICS apresentaram significância estatística. 

 As características da formação em PICS por profissionais de Enfermagem no estado de São Paulo são apresentadas na Figura 1 . As práticas mais identificadas foram a auriculoterapia, a Medicina Tradicional Chinesa/acupuntura e o *Reiki* . Quanto às características dos cursos realizados para formação, prevaleceram as modalidades de curso livre (educação profissional com carga horária reduzida, como oficinas e *workshops* ), presenciais e teórico-práticos. 

 A carga horária representa o número de horas dedicadas à formação teórico-prática em cada curso, oferecendo uma visão geral do tempo necessário despendido para adquirir conhecimentos e habilidades em cada uma das abordagens terapêuticas. Destacam-se a acupuntura, a naturopatia e a terapia comunitária como aquelas com maior carga horária média de formação, e biodança, arteterapia e osteopatia, com menor carga horária. Os valores de médias apresentados na Tabela 4 referentes à carga horária dos cursos em PICS mostraram desvio padrão significativamente alto, indicando que há uma variação considerável na carga horária dos cursos oferecidos, mesmo dentro de uma mesma modalidade de PICS. 

**Tabela 3 -:** Perfil dos profissionais de Enfermagem com formação em Práticas Integrativas e Complementares em Saúde no estado de São Paulo (N* = 3.794). São Paulo, Brasil, 2022

**Variáveis**	**Passo 1**				**Passo 2**				**Passo 3**			
	**RP** ^†^	**I.C.** ^‡^ **(95%)**		**p-valor** ^¶^	**RP** ^†^	**I.C.** ^‡^ **(95%)**		**p-valor** ^¶^	**RP** ^†^	**I.C.** ^‡^ **(95%)**		**p-valor** ^¶^
		**L.I.** ^§^	**L.S.** ^||^			**L.I.** ^§^	**L.S.** ^||^			**L.I.** ^§^	**L.S.** ^||^	
Idade	1,02	1,01	1,03	**0,0001**	1,02	1,01	1,02	**0,0002**	1,01	1,01	1,02	**0,0004**
Sexo feminino	1,23	0,95	1,60	0,1219								
Raça/Cor (Amarelo/Branca/Indígena)	1,21	1,01	1,46	**0,0389**	1,10	0,92	1,32	0,3036				
Renda familiar (Entre 2 e 4 salários**)	1,56	1,12	2,18	**0,0084**	1,08	0,78	1,51	0,6378	1,09	0,79	1,49	0,6048
Renda familiar (Entre 4 e 10 salários**)	3,02	2,21	4,13	**0,0001**	1,30	0,94	1,79	0,1129	1,15	0,84	1,58	0,3680
Renda familiar (Acima de 10 salários**)	4,16	2,90	5,96	**0,0001**	1,44	1,00	2,08	0,0505	1,16	0,82	1,63	0,4105
Religião (Não)	1,27	1,01	1,60	**0,0373**	1,25	1,01	1,56	**0,0416**	1,23	1,02	1,48	**0,0342**
Formação (Superior)					1,97	1,35	2,88	**0,0004**	1,46	1,02	2,09	**0,0404**
Formação ( *Lato sensu* )					4,88	3,54	6,71	**0,0001**	2,77	2,03	3,78	**0,0001**
Formação ( *Stricto sensu* )					7,39	5,16	10,58	**0,0001**	3,32	2,36	4,66	**0,0001**
Carga horária semanal (= 40 horas)					1,57	1,26	1,96	**0,0001**	1,40	1,14	1,71	**0,0013**
Informou saber o que são PICS ^††^ (Sim)									4,84	2,35	10,01	**0,0001**
Vivência ou experiência com as PICS ^††^ (Sim)									10,47	7,18	15,28	**0,0001**

*N = Número de participante analisados

† RP = Razão de prevalência. Foi estimada a probabilidade de apresentar o resultado “Sim”

‡ I.C. = Intervalo de confiança

§ L.I. = Limite inferior

|| L.S. = Limite superior

¶ p-valor = Nível de significância a partir do Modelo de Regressão de Poisson múltiplo modificado hierárquico, com variância robusta

** Salário mínimo vigente = R$ 1.212,00, Brasil, 2022

†† PICS = Práticas Integrativas e Complementares em Saúde

**Figura 1:** - Características autorreferidas dos cursos de formação em Práticas Integrativas e Complementares em Saúde realizados por profissionais de Enfermagem no estado de São Paulo (N* = 479). São Paulo, Brasil, 2022

*N = Número de participantes analisados; ^†^ PICS = Práticas Integrativas e Complementares em Saúde; ^‡^ EAD = Ensino à distância; ^§^ T/P = Teórico/Prático

**Tabela 4:** - Carga horária autorreferida dos cursos de formação em Práticas Integrativas e Complementares em Saúde realizados por profissionais de Enfermagem no Estado de São Paulo (N* = 479). São Paulo, Brasil, 2022

**PICS**	N*	**Média**	**Desvio-padrão**	**Mínimo**	**Q1** ^†^	**Mediana**	**Q3** ^‡^	**Máximo**
Antroposofia	7	167,14	180,44	30,00	40,00	120,00	220,00	540,00
Apiterapia	1	56,00	-	-	-	-	-	-
Aromaterapia	56	159,80	273,83	5,00	20,00	40,00	180,00	1744,00
Arteterapia	1	30,00	-	-	-	-	-	-
Auriculoterapia	165	124,45	211,10	0,00	30,00	75,00	100,00	1300,00
Ayurveda	6	298,67	401,93	12,00	60,00	200,00	220,00	1100,00
Biodança	3	10,00	8,66	5,00	5,00	5,00	20,00	20,00
Constelação	27	232,22	223,69	0,00	80,00	150,00	360,00	900,00
Cromoterapia	22	59,64	77,67	2,00	30,00	40,00	60,00	360,00
Dança Circular	7	94,00	137,77	16,00	20,00	40,00	90,00	400,00
Geoterapia	0	-	-	-	-	-	-	-
Homeopatia	6	400,00	301,70	42,00	58,00	450,00	600,00	800,00
Hipnoterapia	5	42,00	30,92	0,00	26,00	40,00	72,00	72,00
Imposição das mãos	7	64,57	44,22	20,00	24,00	50,00	120,00	120,00
MTC/Acupuntura	125	860,59	584,22	3,00	380,00	800,00	1200,00	3500,00
Meditação	24	94,54	86,94	2,00	30,00	50,00	170,00	300,00
Musicoterapia	4	35,00	32,72	4,00	12,00	28,00	58,00	80,00
Naturopatia	13	748,46	777,49	30,00	460,00	480,00	480,00	3000,00
Osteopatia	1	30,00	-	-	-	-	-	-
Ozonioterapia	31	218,32	382,79	20,00	120,00	120,00	200,00	2200,00
Quiropraxia	1	40,00	-	-	-	-	-	-
Plantas medicinais e fito	17	221,29	214,61	8,00	60,00	120,00	360,00	720,00
Reflexoterapia	19	252,89	458,21	8,00	40,00	100,00	288,00	2000,00
*Reiki*	93	125,37	394,17	1,00	16,00	30,00	60,00	2400,00
*Shantala*	8	39,25	58,38	8,00	8,00	19,00	36,00	180,00
Terapia Comunitária	10	409,80	583,06	8,00	30,00	210,00	500,00	1940,00
Terapia Floral	37	191,73	234,68	4,00	30,00	90,00	360,00	1200,00
Termalismo/Crenoterapia	0	-	-	-	-	-	-	-
Yoga	7	259,14	212,88	14,00	120,00	180,00	500,00	600,00
Outras	74	398,42	655,09	0,00	40,00	135,00	360,00	4138,00

* N = Número de participantes analisados

† Q1 = Primeiro quartil

‡ Q3 = Terceiro quartil

## Discussão

A população com formação em PICS apresentou média de idade de 43,05 anos, com predomínio do sexo feminino, raça/cor branca, amarela e indígena, que vive com parceiro, sem declaração de religião, rendimento entre 4 e 10 salários-mínimos, com formação superior especializada e, em média, 15 anos de formação e experiência profissional. Observou-se uma diferença significativa no que se refere à média de idade e de tempo de formação, e experiência profissional, sendo maior entre a população que tem formação em PICS.

 Quanto ao conhecimento, 39% (n=1481) informaram desconhecimento sobre PICS. Resultado similar foi identificado entre enfermeiras iranianas, cujo conhecimento sobre PICS foi avaliado como baixo ^(^ 17 ^)^ . Um estudo de mapeamento que trabalhou com uma amostragem de conveniência com retorno de dez países europeus não encontrou uma abordagem consistente na formação de enfermeiros em PICS, pois, apesar de serem ministrados cursos em institutos regulares de ensino, eles não são integrados ao ensino regular de graduação ^(^ 18 ^)^ . Embora os achados deste estudo sejam convergentes com o referido estudo, a interpretação dos dados deve considerar as características histórico-culturais dos diferentes países a respeito das racionalidades médicas e sistemas tradicionais de cura, bem como as características das políticas de formação e da atuação dos profissionais nas PICS. 

 No presente estudo, profissionais com formação em PICS mencionaram que as práticas mais frequentes foram auriculoterapia, Medicina Tradicional Chinesa/acupuntura e *Reiki* . Esse resultado está em discordância com os dados da Pesquisa Nacional de Saúde, que indica acupuntura, homeopatia, plantas medicinais, meditação e yoga ^(^ 8 ^)^ . A auriculoterapia como a PICS mais frequente na população estudada pode estar relacionada à capacitação que vem sendo oferecida no Brasil, desde 2016, para profissionais de ensino superior que atuam na APS. Nesses cursos, participam profissionais da Enfermagem do sexo feminino, com média de idade de 36,8 anos ^(^ 19 ^)^ . Esse perfil assemelha-se aos profissionais de Enfermagem do estado de São Paulo investigados no presente estudo. Ao comparar com países europeus, observa-se que as principais PICS ofertadas para a formação em Enfermagem são massagem, meditação, *mindfulness* e técnicas de relaxamento ^(^ 18 ^)^ . 

 A formação, seja em âmbito nacional ou mundial, representa uma lacuna que merece esforços para ampliar o acesso à capacitação dos profissionais de saúde, sobretudo de Enfermagem, atendendo a resultados de investigações internacionais e nacionais que indicam a escassez de conhecimento como uma das principais fragilidades para a ampliação das ofertas das PICS ^(^ 17 ^,^ 20 ^)^ . Esse achado coloca em perspectiva a discussão sobre a formação inicial e continuada dos profissionais de Enfermagem no que se refere às PICS, pois observa-se que falta uma abordagem consistente tanto no Brasil quanto na Europa na educação dos enfermeiros em PICS e Enfermagem integrativa ^(^ 18 ^)^ . Para a Enfermagem europeia, assim como ocorre com a brasileira, são ministrados cursos. Contudo, não há uma diretriz nacional que integre as PICS ao currículo regular de ensino ^(^ 18 ^)^ . 

 Constatou-se, no presente estudo, que os profissionais com alguma formação em PICS também tiveram contato prévio com alguma prática, seja durante a sua formação como profissional ou na instituição onde atuam. Assim, destaca-se a importância de cursos formativos oferecerem disciplinas em seus currículos para sensibilizar os futuros profissionais sobre a temática e apresentar as PICS como uma abordagem de cuidado à saúde. No Brasil, observa-se que as graduações em Enfermagem, Medicina e Odontologia têm como característica a oferta de disciplinas sobre PICS eletivas, teóricas e não integradas às matrizes curriculares ^(^ 21 ^-^ 22 ^)^ . 

 Ainda com relação à formação, os achados refletem a variabilidade na carga horária para cada uma das diferentes formações. Esse resultado corrobora a ausência de diretrizes regulatórias a respeito de um currículo mínimo, com consequentes discrepâncias. Nesse sentido, urge a necessidade de serem estabelecidas resoluções que norteiem a categoria sobre a carga horária mínima para cada capacitação e para cada prática. Os resultados mostram que os profissionais compreenderam como sendo “formação” tanto cursos livres, de difusão, palestras e *workshops* , quanto cursos de capacitação, mostrando ausência de clareza sobre o que representa um processo formativo. A variabilidade na formação é um aspecto preocupante apontado na literatura, constituindo-se em uma fragilidade para a própria área das PICS, para a formação profissional e para os usuários dos serviços de saúde ^(^ 17 ^)^ . 

 Da formação à atuação também há desafios relativos à disputa por espaço entre categorias profissionais, à cultura institucional, e à disponibilidade de tempo e de recursos para a oferta das PICS ^(^ 23 ^)^ . Esses desdobramentos são observados em contratos empregatícios na Enfermagem, que frequentemente não abrangem ou reconhecem a oferta das PICS como parte de suas atribuições profissionais. Também há fragilidades na gestão dos serviços de saúde, o que dificulta a compreensão, o reconhecimento e a oferta de PICS, concretizada na falta de disponibilização de recursos materiais e na divulgação sistematizada. Essa questão reflete diretamente na discrepância entre a oferta real e aquela registrada nos sistemas de informação, evidenciando a problemática do registro subdimensionado das PICS ^(^ 24 ^)^ . 

 Por outro lado, a formação em PICS e a oferta entre os serviços vêm em uma crescente no território nacional ^(^ 8 ^,^ 21 ^-^ 22 ^)^. Essa situação permite identificar os motivos que fazem profissionais de Enfermagem - maior força de trabalho do Sistema Único de Saúde - persistir no investimento para a formação e oferta de PICS nos serviços de saúde. Essa realidade convoca as entidades governamentais e órgãos de classe profissional a analisarem o arcabouço ético-legal e técnico que sustentam as ações. 

 Quanto à qualificação, constatou-se na população investigada que houve procura por qualificações nas 29 terapias complementares dispostas na PNPIC ^(^ 4 ^)^ , contudo, nas Resoluções específicas da Enfermagem, apenas dez práticas estão incluídas como especialidades da profissão ^(^ 7 ^)^ . A dimensão institucional inclui-se nessa problemática, a exemplo da elaboração e da disponibilização de Protocolos Clínicos e Procedimentos Operacionais Padrão que possibilitam a atuação da Enfermagem na oferta de PICS nos serviços de saúde. 

 Ressalta-se que as PICS estão ganhando protagonismo no cenário mundial. Em 2023 ocorreu a Primeira Cúpula Global de Medicina Tradicional, na Índia, paralelamente à reunião interministerial de saúde dos membros do Grupo dos 20 (G20). O objetivo da Cúpula foi registrar o compromisso político dos estados-membros em oferecer às suas populações a Medicina Tradicional baseada em evidências para atender as necessidades de saúde e bem-estar ^(^ 25 ^)^ . Por tratar-se de uma temática universal de saúde priorizada por entidade global, cabe à Enfermagem incorporar tais práticas no exercício de sua profissão. 

Esse estudo tem limitações que se referem aos dados obtidos sobretudo com relação à formação. Observou-se um desvio padrão considerável na carga horária de formação entre os respondentes, indicando que possivelmente palestras foram consideradas pelos profissionais como atividade de caráter formativo. Nesse sentido, sugere-se que em futuros estudos a pergunta investigativa que norteie tal questão seja atrelada à prática exercida em PICS, como por exemplo, “Quantas horas foram investidas na sua formação para que esteja qualificado para aplicar esta PICS?”.

As contribuições do estudo referem-se ao levantamento dos aspectos relacionados à formação e à atuação da Enfermagem na unidade federativa do país que tem a maior concentração de profissionais, respondendo a uma lacuna científica internacional e nacional quanto ao perfil sociodemográfico e profissional da Enfermagem nas PICS. Com base nos achados, argumenta-se sobre a importância de políticas públicas na área da saúde e sua articulação e informação com as políticas de formação de recursos humanos.

## Conclusão

No estado de São Paulo, embora haja um contingente considerável de profissionais de Enfermagem que têm interesse pelas PICS, quase metade dos profissionais desconhecem tais práticas. A formação na área se dá, principalmente, entre enfermeiras em torno de quarenta anos de idade, com especialização e, aproximadamente, quinze anos de experiência profissional.

Destaca-se que diretrizes mínimas com relação à formação devem ser consideradas para a capacitação dos profissionais de Enfermagem em PICS, bem como nas demais profissões da saúde. A padronização é fundamental para a continuidade e a ampliação do acesso da população às PICS, com garantia de profissionais devidamente qualificados para uma oferta segura dos recursos terapêuticos nos diversos cenários institucionais.
